# Aging of the adaptive immune system affects the gut microbiome and systemic levels of vitamin B6

**DOI:** 10.1186/s40168-026-02428-3

**Published:** 2026-06-05

**Authors:** Selina Stahl, Hanna Widmaier, Vadim Sakk, Kodandaramireddy Nalapareddy, Ann-Kathrin Kissmann, Frank Rosenau, Medhanie A. Mulaw, David B. Haslam, Hartmut Geiger

**Affiliations:** 1 Institute of Molecular Medicine, Ulm University, Ulm, Baden-Württemberg 89081 Germany; 2 Department of Internal Medicine I, University Hospital of Ulm, Ulm, Baden-Württemberg 89081 Germany; 3 Division of Experimental Hematology and Cancer Biology, Cincinnati Children’s Hospital Medical Center and Department of Pediatrics, University of Cincinnati College of Medicine, Cincinnati, OH 45229 USA; 4 Institute of Pharmaceutical Biotechnology, Ulm University, Ulm, Baden-Württemberg 89081 Germany; 5 Unit for Single-Cell Genomics, Ulm University, Ulm, Baden-Württemberg 89081 Germany; 6 Microbial Genomics and Metagenomics Laboratory, Cincinnati Children’s Hospital Medical Center and Department of Pediatrics, University of Cincinnati College of Medicine, Cincinnati, OH 45229 USA

**Keywords:** Age-associated gut dysbiosis, Microbiota, Immune system, Aging, Vitamin B6

## Abstract

**Background:**

Age-associated dysregulation of the gut microbiota is a hallmark of aging and has been linked to multiple age-related diseases, yet upstream host factors driving these changes remain incompletely defined. Extensive bidirectional crosstalk between gut microbiota and mucosal immunity has been described. Aging is accompanied by a progressive decline in immune function, collectively termed aging-associated immune remodeling (AAIR). AAIR encompasses widespread compositional and functional changes that impair an effective response to pathogens, vaccines, and tissue damage. We examined whether AAIR is an upstream host factor influencing the composition of the microbiome upon aging.

**Results:**

Hallmarks of AAIR were also present in the ileal lamina propria, including reduced naïve CD4^+^ and CD8^+^ T cell populations and expansion of memory and regulatory T cell subsets. To test whether mucosal AAIR reflects intrinsic aging of the hematopoietic system, we used an HSC transplantation model where young RAG1^−/−^ recipients develop an adaptive immune system derived exclusively from either young or aged donor HSC in an otherwise young host environment. Recipients of aged HSCs recapitulated key features of mucosal AAIR, particularly loss of naïve T cells, demonstrating that AAIR in the ileal LP is driven at least in part by aged HSCs. Shotgun metagenomic sequencing of fecal samples revealed that ileal AAIR is associated with alterations in gut microbiota. In detail, there was a reduced abundance of taxa associated with the vitamin B6 (VB6) biosynthesis and salvage pathways. Accordingly, VB6 levels in serum were reduced in mice with aged immune systems.

**Conclusion:**

Our findings link AAIR to reduced microbial VB6 pathway abundance and lower systemic VB6 availability, suggesting that immune aging shapes the functional output of the microbiome in ways that diminish its VB6 biosynthetic capacity. This postulates an immune-microbiome-VB6 association that warrants further investigations for therapeutic strategies to increase VB6 levels upon aging.

Video Abstract

**Supplementary Information:**

The online version contains supplementary material available at 10.1186/s40168-026-02428-3.

## Background

The gut microbiome supports host health by contributing to nutrient metabolism [1], epithelial barrier integrity [2], immune maturation [3, 4] and function [5], as well as protection against pathogens [6–8]. With aging, the composition of the microbiota undergoes notable shifts, characterized by reduced diversity, increased inter-individual variability [9], and altered functional potential [10]. These age-related changes have been linked to a range of health impairments and diseases commonly associated with aging, such as frailty, chronic inflammation, reduced intestinal regeneration, and neurodegeneration [11–13].

Despite growing recognition of microbial dysbiosis as a driver of aging-associated impairments and diseases, the mechanisms that confer dysbiosis remain elusive. Aging encompasses a wide range of concurrent changes that can individually or collectively shape the gut microbiome [14]. Intrinsic factors include physiological processes such as immune remodeling, hormonal shifts [15], and declining epithelial function [16], while extrinsic influences range from dietary changes [17] and medication use [18] to varying levels of microbial exposure through housing or environment [19]. These overlapping variables make it challenging to disentangle cause from consequence when studying age-related microbiome alterations [19, 20].

The adaptive branch of the immune system plays a central role in maintaining microbial homeostasis, primarily through immunoglobulin A (IgA) secretion [21–23], T cell-mediated immune surveillance [24, 25], and support of epithelial barrier function [26, 27]. Mice with genetic deficiencies in adaptive immune components—such as recombination activation gene 1-deficient (RAG1^−/−^) mice, which lack mature B and T cells—display a significantly altered microbiota composition compared to wild-type mice [28].

Aging of the immune system—referred to as aging-associated immune remodeling (AAIR) [29]—is characterized by a decline in naïve lymphocyte output [30], accumulation of memory and regulatory T cells, thymic involution [31], and reduced immune responsiveness [32, 33]. Aging leads to intrinsic changes in hematopoietic stem cells (HSCs) which then confer this shift toward myeloid-biased differentiation at the expense of lymphoid lineages [29, 34]. AAIR has been primarily identified in the blood and central hematopoietic organs [29]; however, its presence in tissue-resident immune systems—such as the one located in the gut lamina propria (LP), which is in direct contact with the microbiota—remains poorly characterized. Similarly, the extent to which AAIR influences the composition of the gut microbiome is still not well understood.

We hypothesized that aging of the adaptive immune system contributes to the microbiome alterations observed during aging. We employed a HSC transplantation model that results in either a young or aged adaptive immune system in genetically identical, age-matched hosts under standardized housing and dietary conditions—thus enabling the study of the effects of an aged adaptive immune system without other confounding factors [29].

Our results demonstrate that intrinsic immune aging, driven by aged HSCs, contributes to age-associated remodeling of adaptive immune cells in the ileal LP and impacts the gut microbiota. Specifically, mice with an aged adaptive immune system displayed a reduced metagenomic abundance of VB6 biosynthesis and salvage pathways. Notably, these microbial changes coincided with lower host serum VB6 levels. While our data do not establish a direct causal relationship between reduced microbial VB6 metabolic potential and systemic availability, the observed co-occurrence is consistent with a model in which adaptive immune aging may influence microbiome-associated VB6 supply. Given the essential role of VB6 in immune regulation [35, 36], energy metabolism [37], and neuronal health, this association warrants further mechanistic investigation as a potential contributor to age-related physiological decline.

## Methods

### Mice and housing conditions

B6.129S7-Rag1^tm1Mom^/J (RAG1^−/−^) female mice (2–3 months old) were used as recipient mice for HSC transplantation. HSC donor mice consisted of young (2–3 months) and old (>20 months) congenic B6.SJL-*Ptprc*^*a*^*Pepc*^*b*^/BoyJ (B6.SJ) female mice, as previously published [29]. To serve as a reference for age-related immune and microbiome alterations, young (2–3 months) and old (>20 months) female C57BL/6J mice were included in the study.

All animals were housed under specific pathogen-free (SPF) conditions at the Animal Facility of Ulm University (Ulm, Germany). Mice were either bred in-house or obtained from Jackson Laboratories (Bar Harbor, USA). To minimize environmental influences on the gut microbiome, animals were provided with autoclaved food and water, and all procedures (e.g., blood sampling, fecal collection) were conducted in a BSL-2 biosafety cabinet. Bedding was interchanged between cages twice a week preceding fecal sample collection.

All experiments were conducted in compliance with the German Federal Animal Protection Law and the Guide for the Care and Use of Laboratory Animals. All mice experiments were approved by the RP Tübingen (Protocol Numbers: 35/9185.81-3.81/1172).

### HSC isolation and sorting

HSCs were isolated from the bone marrow (BM) of the femur and tibia of young (2–3 months) and old (>20 months) B6.SJL mice. Mononuclear cells (MNCs) were separated from total BM by low-density centrifugation using Ficoll-Paque Premium 1.084 (Cytiva, Marlborough, MA, USA). To enrich for lineage-negative (Lin^−^) cells, MNCs were first stained with a cocktail of biotinylated rat anti-mouse lineage antibodies, including anti-CD11b, anti-B220, anti-CD5, anti-Gr-1, anti-Ter119, and anti-CD8a (Suppl. Table 1). Lineage depletion was performed using magnetic separation (Dynabeads; Invitrogen, Carlsbad, CA, USA). Following depletion, cells were stained with anti-Sca-1, anti-c-Kit, anti-CD34, anti-Flk2, and Streptavidin (all from eBioscience, Waltham, MA, USA). HSCs were isolated as Lin^−^ Sca-1^+^ c-Kit^+^ CD34^−^ Flk2^−^ cells using a BD FACS Aria III (BD Biosciences, San Jose, CA, USA).

### Non-competitive HSC transplantation

Sorted HSCs were transferred into 1.5-mL reaction tubes and cultured for 16 h in Hanks’ Balanced Salt Solution (HBSS; Lonza, Basel, Switzerland) supplemented with 10% fetal bovine serum (FBS; Thermo Scientific, Waltham, MA, USA) at 37 °C, 5% CO_2_, and 3% O_2_. Subsequently, 600 HSCs were suspended in a maximum volume of 200 µL PBS (Gibco, Waltham, MA, USA) and transplanted via intravenous injection into the lateral tail vein of sublethally irradiated (6.5 Gy) RAG1^−/−^ recipient mice on the same day as irradiation. Following transplantation, reconstituted mice were maintained on Neomycin (1.1 mg/mL) in drinking water until 12 weeks post-transplantation. Donor contribution in peripheral blood (PB) was assessed by flow cytometry every 6 weeks, from week 6 to week 18 post-transplantation, using samples collected from the vena facialis.

### Flow cytometric analyses of peripheral blood cells

For immune system characterization, PB immunostaining was performed according to standard protocols. Briefly, erythrocytes from single-cell PB suspensions were lysed with a hypotonic buffer (BioLegend, San Diego, CA, USA). Cells were then incubated with fluorochrome-conjugated antibodies for 20 min at 4 °C.

Samples were acquired using an LSRFortessa Flow Cytometer (BD Biosciences), and data analysis was performed using BD FACSDiva 8.1. All antibodies used for flow cytometry are summarized in Suppl. Table 1.

### Ileal lamina propria immune cell preparation and CyTOF analysis

For characterization of the ileal immune system, the entire gastrointestinal (GI) tract was collected from each mouse. The ileum was defined as the distal portion of the SI extending from the jejuno–ileal transition to the ileocecal junction. This terminal segment was excised and typically measured ~10–12 cm in length, depending on body size. Ileal tissue was then subjected to mechanical and enzymatic digestion to generate a single-cell suspension using the Lamina Propria Dissociation Kit (Miltenyi Biotec, Bergisch Gladbach, Germany) according to the manufacturer’s instructions. Where indicated, cells were stimulated for 5 h at 37 °C in HBSS supplemented with cell stimulation cocktail containing the protein transport inhibitors brefeldin A and monensin (500×; eBioscience) prior to staining. Immunostaining with metal-labeled antibodies was performed following standard protocols (Standard BioTools, South San Francisco, CA, USA). Briefly, erythrocytes were lysed using a hypotonic buffer (BioLegend). Cells were first stained with Cell-ID Cisplatin-195Pt (Standard BioTools) for 5 min, followed by surface staining with metal-labeled antibodies (Standard BioTools) for 30 min at room temperature (RT). For intracellular staining of cytoplasmic and nuclear antigens, cells were permeabilized using Maxpar Nuclear Antigen Staining Buffer (Standard BioTools) for 30 min at RT. The antibody-labeled single-cell suspension was then resuspended in freezing medium (FBS + 10% DMSO) and stored at −80 °C until acquisition on a Helios Mass Cytometer (Standard BioTools). Data analysis was performed using Cytobank Cytometry Software (version 10.6.2.8). All metal-labeled antibodies used for CyTOF are listed in Suppl. Table 2. Antibodies not included in the Maxpar Mouse Spleen/Lymph Node Phenotyping Panel Kit were self-labeled according to the manufacturer’s instructions using the Maxpar MCP9 and X8 Antibody Labeling Kits (Standard BioTools).

### Serum vitamin B6 measurement by competitive ELISA

Blood from young and old C57BL/6J and transplanted RAG1^−/−^ mice was collected via cardiac puncture into serum tubes and incubated for 2 h at RT. Samples were then centrifuged at 10,000 × g for 5 min. The resulting serum, located above the gel barrier, was carefully collected using a 100-µL pipette and transferred into 1.5-mL Eppendorf tubes (Eppendorf SE, Hamburg, Germany), then stored at −80 °C until analysis.

VB6 levels were determined by a competitive inhibition ELISA (Biomatik Corporation, Kitchener, Ontario, Canada), according to the manufacturer’s instructions. Briefly, 50 µL of standards and samples (in duplicates) were added to microplate wells pre-coated with anti-VB6 antibodies. Following a 1-h incubation at 37 °C with biotin-labeled VB6 (Detection Reagent A), unbound conjugates were removed by washing. Avidin-HRP was added and incubated for 30 min at 37 °C. After substrate addition, absorbance was measured at 450 nm using a Varioskan LUX microplate reader (Thermo Scientific). The intensity of the colorimetric signal was inversely proportional to the VB6 concentration in the samples.

### Ileal secretory IgA measurement by competitive ELISA

Ileal contents from Y and O C57BL/6J mice were collected after harvesting the ileum as described above. Briefly, the ileum was opened longitudinally and luminal contents were collected using sterile forceps. Samples were resuspended in PBS (Gibco) at a ratio of 500 mg ileal content per 2 mL PBS, adjusted proportionally according to the exact sample weight, and stored at −80 °C until analysis. sIgA levels were determined using the colorimetric Mouse Secretory IgA ELISA Kit (Novus Biologicals, Centennial, CO, USA), according to the manufacturer’s instructions. Briefly, 100 µL of standards and samples (in duplicates) were added to the appropriate microplate wells and incubated for 90 min at 37 °C. After addition of biotinylated detection antibody and incubation for 1 h at 37 °C, wells were washed and incubated with HRP conjugate for 30 min at 37 °C. Following substrate addition, absorbance was measured at 450 nm using a Varioskan LUX microplate reader (Thermo Scientific). Concentrations were calculated based on a four-parameter logistic standard curve.

### *Ephb6* gene expression analysis by qPCR

Peripheral blood mononuclear cells (PBMCs) were isolated from whole blood of Y and O C57BL/6J mice by red blood cell lysis (5 min, RT) using a hypotonic buffer (BioLegend). Ileal LPLs were prepared as described above, and CD4^+^ T cells were purified by cell sorting using a BD FACSAria III cell sorter (BD Bioscience). Total RNA was extracted from PBMCs and sorted CD4^+^ ileal LPLs using the RNeasy Micro Kit (Qiagen, Hilden, Germany) according to the manufacturer’s instructions. RNA concentration was determined using a NanoPhotometer P330 (Implen, Munich, Germany). To ensure comparability across samples, equal RNA input was used for cDNA synthesis. Reverse transcription was performed using the QuantiTect Reverse Transcription Kit (Qiagen) following the manufacturer’s protocol.

qPCR was performed in technical triplicates for each sample using TaqMan Gene Expression Assays (Thermo Scientific) for *Ephb6* (Assay ID: Mm00432456_m1) and the reference gene *Gapdh* (Assay ID: Mm99999915_g1). Reactions were run according to the manufacturer’s recommendations (Thermo Scientific). Cycle threshold (Ct) values were obtained using automatic baseline and threshold settings. Relative expression was calculated using the comparative Ct method (2^−ΔΔCt) [38]. Fold change relative to the mean of the young group was reported as 2^−ΔΔCt. For statistical analysis, ΔCt values were compared between Y and O groups using an unpaired Welch’s *t*-test.

### Microbiota sampling, DNA extraction, and shotgun metagenome sequencing

Fecal microbiota samples were collected individually from each mouse. Fresh fecal pellets were retrieved directly from the anus using sterile forceps and placed into pre-weighed, sterile tubes. Sample collection and handling were conducted under a sterile laminar flow hood to minimize contamination. All samples were immediately stored at −80 °C until further processing.

Genomic DNA was extracted from one or two fecal pellets using the PowerFecal DNA Isolation Kit (MO BIO Laboratories, Carlsbad, CA, USA), following the manufacturer’s instructions. DNA concentration was quantified using a Qubit fluorometer (Thermo Scientific). Library preparation was performed using the Nextera XT protocol (Illumina, Inc., San Diego, CA, USA) according to the manufacturer’s guidelines. Shotgun metagenomic sequencing was conducted on an Illumina NovaSeq X platform, generating 150 bp paired-end reads to a depth of approximately 20 million reads per sample.

### Metagenomic data analysis

Raw sequence reads were extracted and demultiplexed using the Illumina program bcl2fastq (v2.20.0). Raw reads were filtered and trimmed for quality control using Sickle [39]. Taxonomic classification was performed using Kraken2 (v2.1.2) with the standard Kraken2 database (downloaded from https://benlangmead.github.io/aws-indexes/k2), which includes RefSeq complete bacterial, archaeal, and viral genomes, as well as the human genome (GRCh38) for host read filtering [39]. Species-level abundances were refined using Bracken (v2.6.2) with a read length of 150 bp and a minimum classification threshold of 10 reads. Functional profiling of microbial pathways was performed using HUMAnN3 (v3.9) with the MetaCyc pathway database. Pathway abundances (representing gene family copy number) were normalized to copies per million (CPM) as provided by HUMAnN3.

#### Community structure, diversity, and differential taxonomic abundance

Species with fewer than 0.001% of total reads or present in fewer than 5% of samples were removed. Samples with fewer than 750,000 total classified reads were excluded, and remaining count data were rarefied to a uniform depth of 750,000 reads using the rrarefy function from the vegan package (v2.7-2) in R [40]. Rarefaction curves plateaued well before 750,000 reads for most samples analyzed, indicating that sequencing depth was sufficient to capture the majority of detectable diversity in each community. Principal component analysis (PCA) was performed on generalized log_2_-transformed species abundance data using the FactoMineR package (v2.12) in R, with visualizations generated using the factoextra package (v1.0.7). Overall differences in microbiome composition between groups were assessed using multiresponse permutation procedures (MRPP) with Bray-Curtis distance, implemented in the vegan package [41]. Differential species and genus abundance between groups was determined by pairwise Wilcoxon rank-sum tests. Where indicated in the “Results”, *p *values were adjusted for multiple testing using either Bonferroni correction (for α-diversity comparisons) or the Benjamini-Hochberg false discovery rate (FDR) procedure (for feature-level comparisons). Fold changes and log_2_ fold changes (Log_2_FC) were calculated using the gtools package (v3.9.5) in R. Alpha diversity was assessed using three complementary metrics: Shannon index (richness and evenness), Simpson index (probability of interspecific encounter), and Chao1 estimator (species richness including unobserved species), calculated using the vegan package (v2.7-2) in R.

#### Pathway differential abundance analysis

Global differential abundance analysis of MetaCyc pathways (HUMAnN3 CPM-normalized gene content) between experimental groups was performed using pairwise Wilcoxon rank-sum tests without multiple testing correction as an initial hypothesis-generating screen. Two VB6-associated pathways consistently identified in this exploratory screen—PYRIDOXSYN-PWY (pyridoxal 5′-phosphate biosynthesis I) and PWY0-845 (superpathway of pyridoxal 5′-phosphate biosynthesis and salvage)—were subjected to targeted reanalysis. Experimental batch effects were incorporated as a covariate using ALDEx2 (v1.40.0), a compositionally aware differential abundance framework for sparse, zero-inflated microbiome count data. ALDEx2 performs centered log-ratio (CLR) transformation of Monte Carlo-sampled Dirichlet-multinomial probabilities (128 instances per sample) to account for compositional structure and library size variation. Differential abundance was tested using the generalized linear model function aldex.glm with design matrix ~ Experiment + Group, where “Experiment” models technical batch variation across sequencing runs and “Group” tests the primary biological comparison. Effect sizes are reported as CLR mean differences between groups. *p *values were adjusted for multiple testing across pathways using the Benjamini-Hochberg FDR procedure (threshold FDR < 0.05).

#### Power analysis

Post hoc power analysis was performed for primary between-group comparisons of alpha-diversity metrics using the pwr package (v1.3.0) in R. Cohen’s *d* effect sizes were calculated from observed Shannon, Simpson, and Chao1 values between experimental groups. Achieved statistical power (1 − *β*) was estimated using a two-sample *t*-test framework at *α* = 0.05. Sample sizes required to achieve 80% power (1 − *β* = 0.80) at the observed effect sizes were calculated and reported.

## Results

### Adaptive immunity and age influence the composition of the gut microbiome

**Fig. 1:**
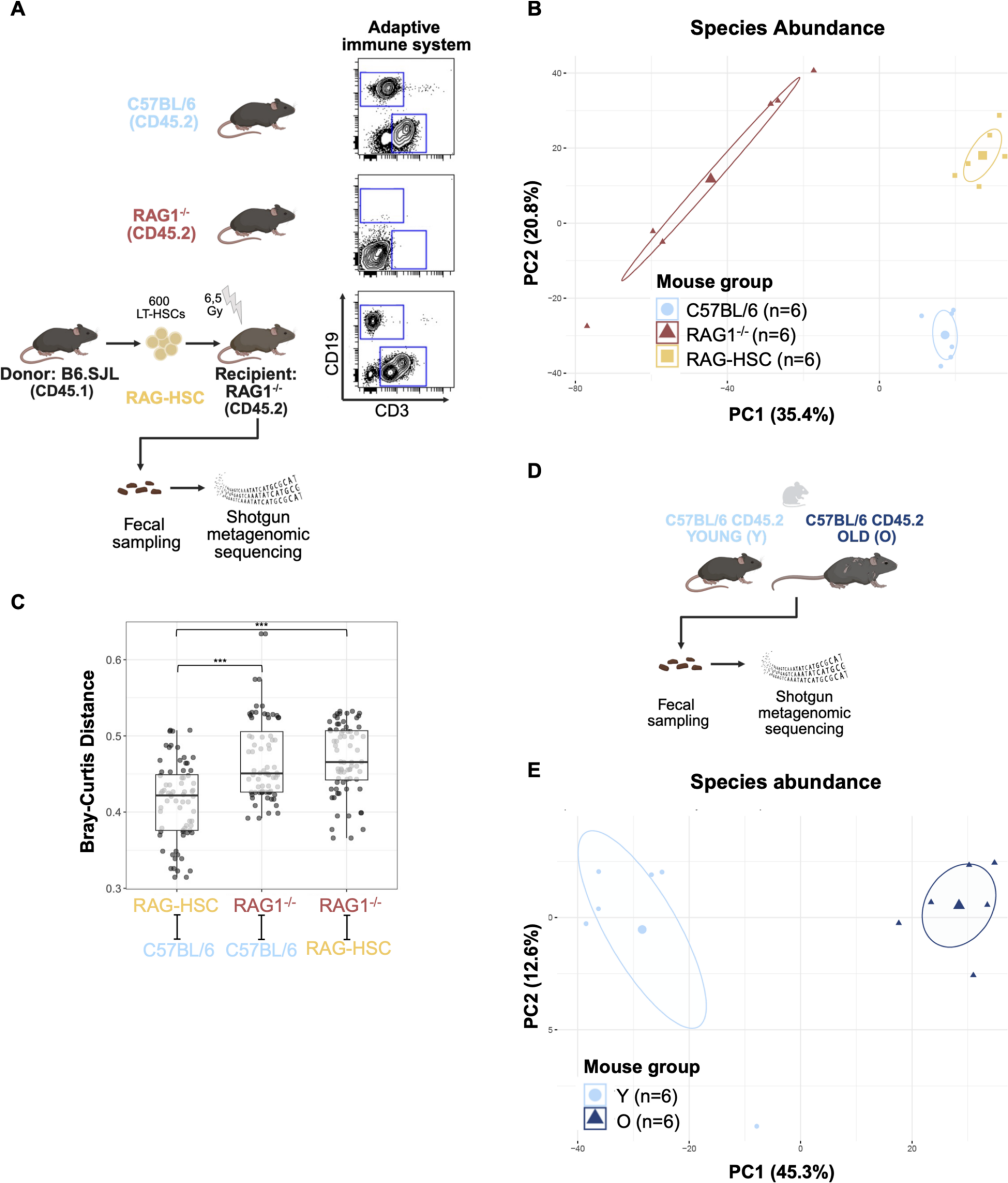
Adaptive immunity and age influence the composition of the gut microbiome. **A** Schematic of the experimental setup. Fecal samples were collected from wild-type C57BL/6J mice (light blue), RAG1^−/−^ mice (red), and RAG-HSC mice (yellow). RAG-HSC mice were generated by transplanting 600 hematopoietic stem cells (HSCs) from B6.SJL (CD45.1^+^) donors into sublethally irradiated (6.5 Gy) RAG1^−/−^ (CD45.2^+^) recipients. RAG1^−/−^ mice lack adaptive immune cells, whereas C57BL/6J and RAG-HSC mice possess functional B and T cells. Shotgun metagenomic sequencing was performed on all groups. **B** Principal component analysis (PCA) of centered log-ratio (CLR)–transformed species-level microbial abundances, showing distinct clustering by mouse group. PC1 and PC2 explained 35.4% and 20.8% of the total variance, respectively. Samples are color-coded by group: C57BL/6J (*n* = 6), RAG1^−/−^ (*n* = 6), and RAG-HSC (*n* = 6). Ellipses indicate the 95% confidence regions for each group. **C** Bray-Curtis distance comparisons between groups, illustrating microbial community dissimilarities among C57BL/6J, RAG1^−/−^, and RAG-HSC mice. *p *values for pairwise comparisons: *p*_1_ ≤ 0.001; *p*_2_ ≤ 0.001. **D** Schematic of the experimental setup. Fecal samples were collected from young (Y; light blue) and old (O; dark blue) C57BL/6J mice, followed by shotgun metagenomic sequencing. **E** PCA of CLR-transformed species-level microbial abundances, showing distinct clustering by age group. PC1 and PC2 explained 45.3% and 12.6% of the total variance, respectively. Samples are color-coded by group: Y (*n* = 6; 9 weeks old, median age = 9) and O (*n* = 6; 86–89 weeks old, median age = 89). Statistical significance was assessed using the multiresponse permutation procedure (MRPP) for PCA clustering and Dunn’s test for pairwise comparisons of Bray-Curtis distances. Data in **C** is presented as box plots showing the 0.25, 0.5 (median), and 0.75 percentiles, with whiskers indicating minimum and maximum values. Asterisks indicate statistical significance: ***p < 0.001

To determine the influence of adaptive immunity in shaping the overall composition of the gut microbiome [28], we analyzed fecal microbial distribution using shotgun metagenomic sequencing from three groups of mice: (i) RAG1-deficient (RAG1^−/−^) mice, which lack functional B and T cells due to impaired V(D)J recombination; (ii) immunocompetent C57BL/6J wild-type controls; and (iii) RAG-HSC mice, generated by transplanting hematopoietic stem cells (HSCs) from congenic CD45.1^+^ B6.SJL donors into sublethally irradiated RAG1^−/−^ recipients, thereby reconstituting functional adaptive immunity in the host [29] (Fig. 1A).

Principal component analysis (PCA) based on centered log-ratio (CLR) transformed species-level abundance revealed distinct clustering of microbial communities in RAG1^−/−^ mice (red) compared to wild-type controls (C57BL/6J; light blue), while RAG-HSC mice (yellow) clustered separately but in closer proximity to wild-type animals (Fig. 1B). Bray-Curtis distance analysis confirmed that the microbial profiles of RAG-HSC mice were significantly more similar to those of wild-type mice than to unreconstituted RAG^−/−^ controls (Fig. 1C). To quantify within-sample diversity, we calculated three complementary α-diversity metrics (Shannon, Simpson, and Chao1) for C57BL/6J, RAG1^−/−^, and RAG-HSC mice (Suppl. Fig. 1A–C). Shannon and Simpson indices were significantly higher in non-transplanted RAG1^−/−^ mice than in both C57BL/6J and RAG-HSC mice, implying that adaptive immunity imposes indeed a selective filter on the microbiota and thereby constrains community evenness (Suppl. Fig. 1A, B). In contrast, Chao1 richness was highest in C57BL/6J mice and lower in RAG1^−/−^ mice, while RAG-HSC mice did not differ significantly from RAG1^−/−^ mice (Suppl. Fig. 1C), indicating that group differences are not explained solely by richness and are likely driven to a substantial extent by differences in evenness/community structure.

These findings indicate that the absence of adaptive immunity in RAG1^−/−^ mice is associated with an altered gut microbiome composition compared to immunocompetent wild-type controls. Reconstitution of adaptive immunity via HSC transplantation partially shifted the microbial community structure toward that of wild-type mice, with intermediate α-diversity values consistent with partial restoration of immune-mediated microbial selection. This supports the notion that adaptive immunity contributes to the modulation and maintenance of a balanced intestinal microbiota. This observation aligns with and extends previous studies, underscoring the role of adaptive immune status in shaping gut microbial communities [28].

In a next step, we assessed aging-associated changes in the gut microbiome composition in mice housed within our facility, as such changes reported in the literature remain variable and are thought to be strongly influenced by environmental factors such as housing, diet, and microbial exposure [42]. To this end, we performed shotgun metagenomic sequencing of fecal samples from young (Y, 2–3 months) and old (O, >20 months) C57BL/6J mice (Fig. 1D). PCA of CLR-transformed species-level abundances revealed clear separation between Y and O mice, indicating age-dependent differences in overall community structure (Fig. 1E). Consistent with previous studies comparing the microbiomes of young and aged mice, we observed, among other changes, a trend toward an age-associated increase in the Firmicutes-to-Bacteroidota ratio [43] (Suppl. Fig. 1D) and a reduction in *Akkermansia muciniphila* abundance [44, 45] (Suppl. Fig. 1E). Effect size analysis further identified shifts in the relative abundance of additional microbial species, including taxa such as *Romboutsia ilealis* and *Bacteroides uniformis*, which have not previously been associated with aging in mice (Suppl. Fig. 1F). These results demonstrate that aging alters the composition of the gut microbiome in our experimental setting in ways that are consistent with earlier reports. In addition to these established patterns, we also detected changes that have not been reported before, which may either reflect environment-specific effects or represent more general, yet previously unrecognized, features of microbial aging.

### Aging-associated remodeling of the ileal immune landscape is accompanied by changes in T-cell subsets

Changes in the composition of both the innate and adaptive immune systems are well-established features of AAIR. These compositional shifts are well-characterized in peripheral immune compartments such as blood and spleen [29, 46], where they define a consensus set of age-related immune alterations. In line with these established AAIR patterns, aged mice housed in our facility showed similar changes in PB compared to their young counterparts (Suppl. Fig. 2A–G). These included a reduced frequency of B cells (Suppl. Fig. 2B), a decrease in the proportion of naïve CD4^+^ and CD8^+^ T cells (Suppl. Fig. 2D, E), and a corresponding increase in memory T cell populations (Suppl. Fig. 2F, G). Alongside these adaptive immune changes, aged mice also exhibited elevated levels of myeloid cells, including granulocytes and monocytes/macrophages (Suppl. Fig. 2C).

**Fig. 2:**
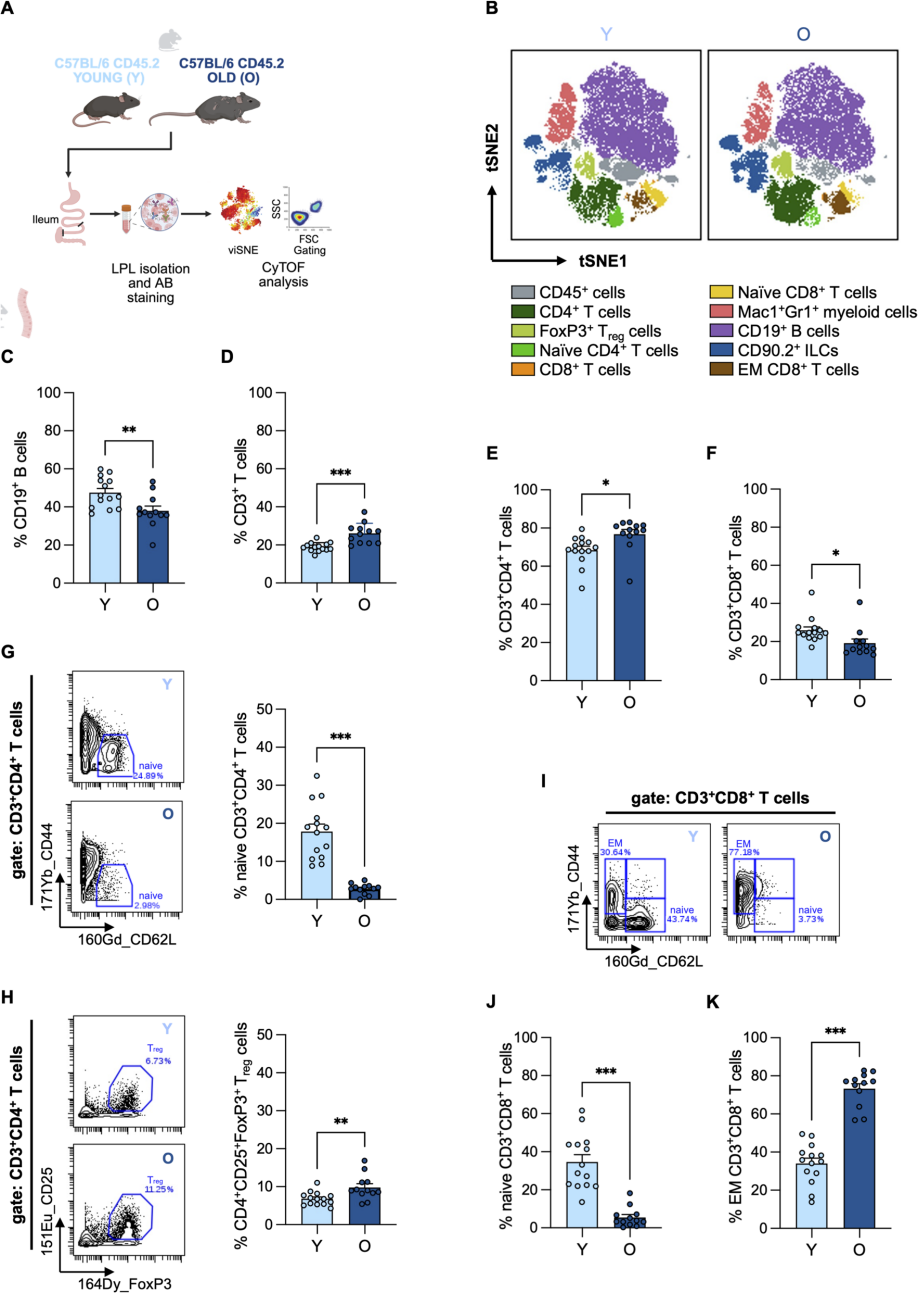
Aging-associated remodeling of the ileal immune landscape is accompanied by changes in T-cell subsets. **A** Schematic of the experimental setup. Ileal tissue was collected from Y (light blue) and O (dark blue) C57BL/6J mice. Lamina propria lymphocytes (LPLs) were isolated and analyzed via CyTOF. **B** The viSNE algorithm was applied to CD45^+^ ileal LPLs to visualize the immune landscape in Y and O mice. Representative viSNE plots of Y (left) and O (right) mice are shown, with color-coded immune cell subpopulations. **C**–**K** Quantification of immune cell subsets of LPLs from Y (*n* = 14; 12–18 weeks old, median = 13) and O (*n* = 12; 84–93 weeks old, median = 92) mice was performed using standard gating: **C** Frequency of CD19^+^ B cells (*p* = 0.007) and **D** CD3^+^ T cells (*p* < 0.001) within CD45^+^ ileal LPLs. **E** Frequency of CD4^+^ T cells (*p* = 0.013) and **F** CD8^+^ T cells (*p* = 0.022) within CD3^+^ T cells of the ileal LP. **G** Representative dot plots and quantification of CD44^−^CD62L^+^ naïve CD4^+^ T (T_N_) cells (*p* < 0.001), and **H** CD25^+^FoxP3^+^ regulatory T (T_reg_) cells (*p* = 0.001), within CD4^+^ T cells of the ileal LP. **I** Representative dot plots showing the gating strategy for CD44^−^CD62L^+^ T_N_ and CD44^+^CD62L^−^ effector memory (T_EM_) CD8^+^ T cells. **J** Frequency of CD44^−^CD62L^+^ CD8^+^ T_N_ cells (*p* < 0.001) and **K** CD44^+^CD62L^−^ T_EM_ CD8^+^ T cells (*p* < 0.001) within CD8^+^ T cells of the ileal LP. Statistical significance was assessed using unpaired Student’s *t*-test (**C**–**G**, **J**–**K**). Data are presented as mean ± SD. Asterisks indicate statistical significance: *p < 0.05; **p < 0.01; ***p < 0.001

Whether such age-related changes in immune cell composition also affect the gut mucosa remains less understood. Thus, to determine whether AAIR also extends to the intestinal mucosa, we analyzed the immune composition of the ileal LP. The ileum is distinguished by high immune activity and exposure to a relatively dense microbial population [47] and, importantly, since it is a major site of nutrient absorption [48, 49], age-related changes here might systemically influence the host. These characteristics make the ileum a critical location for studying AAIR at a mucosal barrier site. We therefore confined our study to this region. Immune cell composition varies considerably along the GI tract [45, 50, 51] and examining a defined site avoids masking age-related changes due to regional differences. LP cells were isolated from the ileum of Y and O mice and analyzed by mass cytometry (CyTOF) to determine the frequency of distinct types of immune cells. viSNE projections were generated based on CD45^+^ cells—referred to here as lamina propria lymphocytes (LPLs)—to exclude non-immune gut-resident cells such as epithelial and stromal populations (Fig. 2A/B). Our analyses revealed several age-associated changes in the relative abundance of immune cell subsets, with particularly pronounced alterations in the abundance of CD19^+^ B cells and CD3^+^ T cell subpopulations—including both CD4^+^ and CD8^+^ subsets (Fig. 2B).

Quantification of the percentage of cell populations by standard gating revealed a reduced frequency of CD19^+^ B cells among CD45^+^ LPLs in aged mice (Fig. 2C), accompanied by an increased frequency of CD3^+^ T cells (Fig. 2D). Although Gr-1^+^ and Mac-1^+^ granulocytes and monocytes/macrophages were elevated in the PB of aged mice (Suppl. Fig. 2C)—consistent with the well-documented myeloid bias during aging [52–54]—their relative abundance within ileal LPLs remained unchanged (Suppl. Fig. 2H). The absence of a myeloid bias in the ileal mucosal compartment suggests tissue-specific regulation of myeloid populations and highlights the need for further investigation into differential immune remodeling across systemic and mucosal sites.

Within the T cell compartment, the frequency of CD4^+^ T cells among CD3^+^ LPLs was elevated in O mice (Fig. 2E), while CD8^+^ T cell frequencies were reduced (Fig. 2F). A central hallmark of AAIR—the reduction of the frequency of naïve T (T_N_) cells [29, 55]—was also evident in aged ileal LPLs, where both CD4^+^ (Fig. 2G) and CD8^+^ (Fig. 2J) T_N_ cell subsets were significantly decreased compared to those in young mice. Conversely, the frequency of CD8^+^ effector memory T (T_EM_) cells was increased in aged ileal CD8^+^ T cells (Fig. 2K), reflecting a shift toward a more antigen-experienced T cell phenotype, consistent with observations in PB from old mice (Suppl. Fig. 2D–G). In parallel, the frequency of CD4^+^CD25^+^Foxp3^+^ regulatory T (T_reg_) cells was also elevated in aged LPLs (Fig. 2H), suggesting an altered immunoregulatory environment in the aged ileal LP.

To determine whether this age-associated remodeling of the ileal immune landscape was also accompanied by functional alterations, we next assessed mucosal immune readouts in Y and O mice. As a measure of immune effector function at the luminal interface, secretory IgA (sIgA) was quantified in ileal contents by ELISA. Ileal sIgA levels showed a trend toward lower abundance in aged mice relative to young controls (Suppl. Fig. 3A), suggesting a possible reduction in mucosal antibody output in the aged ileum. This observation is consistent with the reduced frequency of B cells observed in the aged ileal LP (Fig. 2C) and may reflect impaired local humoral immunity. Because sIgA is a key mediator of host–microbiota interactions, this trend may also contribute to age-associated alterations in microbial community composition.

We also examined cytokine production by ileal CD4^+^ T cells after ex vivo stimulation with PMA/ionomycin in the presence of protein transport inhibitors. We focused on CD4^+^ T cells because this compartment showed marked age-associated phenotypic remodeling in the ileal LP. The frequencies of TNF-α^+^ and IL-2^+^ CD4^+^ T cells were significantly lower in aged mice than in young controls (Suppl. Fig. 3B, D), indicating reduced responsiveness of aged mucosal CD4^+^ T cells to stimulation. In addition to the frequency of cytokine-positive cells, we assessed cytokine signal intensity within the responding populations as a proxy for per-cell cytokine abundance. TNF-α signal intensity showed a trend toward higher values in young mice, whereas IL-2 signal intensity was comparable between groups (Suppl. Fig. 3C, E). IL-6, IL-10, IFN-γ, and IL-17A were also assessed, but did not show age-associated differences. These data imply that aged LP CD4^+^ T cells display diminished responsiveness to stimulation with respect to increasing levels of TNF-α and IL-2, which is consistent with an age-associated decline in adaptive immune function.

Taken together, these findings demonstrate that the composition of immune cells in the gut mucosa, particularly within the ileum, changes markedly with age. Hallmarks of AAIR—such as reduced B cell frequencies, loss of T_N_ cells, and increased T_reg_ frequencies—are not limited to blood and spleen, but are also clearly evident in the ileal LP. Importantly, these compositional changes are accompanied by functional alterations, including a trend toward reduced mucosal sIgA levels and diminished TNF-α and IL-2 responses in ileal CD4^+^ T cells, consistent with declining adaptive immune function at the intestinal barrier.

### Aged HSCs induce aging-associated T cell–specific remodeling in the ileal LP

HSCs reside primarily in the BM and give rise to the majority of immune cells in all peripheral hematopoietic tissues. AAIR in blood, spleen, and BM is to a large extent driven by aged HSCs [29]. Aged HSCs exhibit altered differentiation potential, which underlies many of the immune compositional changes characteristic of AAIR [56]. Immune cells in these tissues are therefore continuously replenished through HSC-derived differentiation, with only a small proportion maintained independently of this process. In contrast, the extent to which BM–resident HSCs contribute to immune cell turnover in the ileum remains unclear. Consequently, both the extent of AAIR and whether the features of AAIR observed in the ileum, as described above, are also a consequence of HSC aging, is not known in detail.

**Fig. 3:**
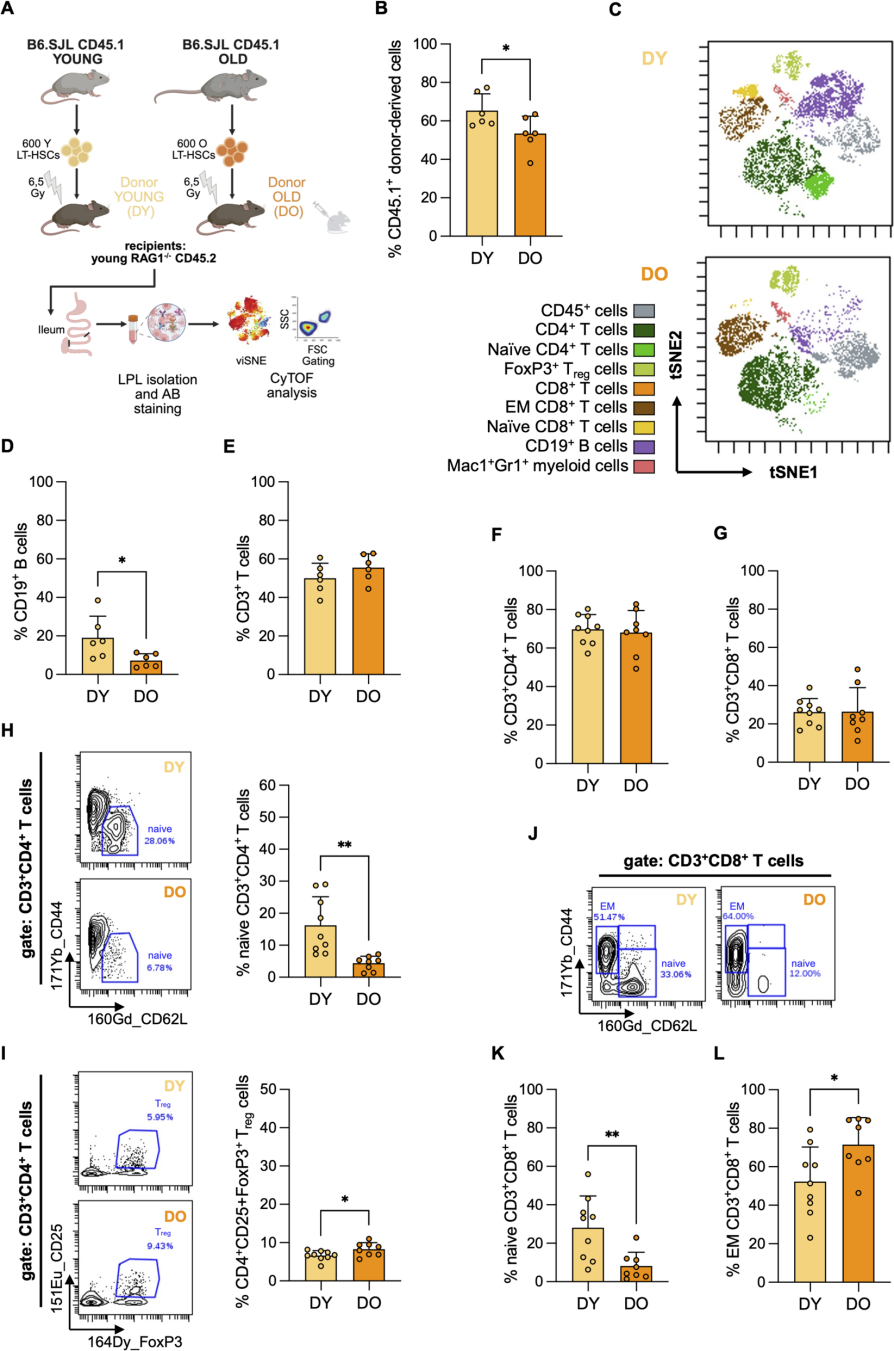
Aged HSCs induce aging-associated T cell–specific remodeling in the ileal LP. **A** Schematic of the experimental setup. Six hundred HSCs were isolated from young and old B6.SJL (CD45.1^+^) donors and subsequently transplanted into irradiated young RAG1^−/−^ (CD45.2^+^) recipients. Recipient mice were designated as DY (yellow) or DO (orange) based on the age of their respective HSC donor. Twenty weeks post-transplantation ileal tissue was collected from DY and DO recipient mice. LPLs were isolated and analyzed via CyTOF. **B** Donor contribution (CD45.1^+^) to total CD45^+^ (CD45.1^+^ and CD45.2^+^) ileal LPL population was assessed in DY (*n* = 6; 11–23 weeks, median = 15) and DO (*n* = 6; *n* = 11–22 weeks, median = 11) mice (*p* = 0.042). **C** The viSNE algorithm was applied to CD45.1^+^ ileal LPLs to visualize the donor-derived immune landscape in DY and DO mice. Representative viSNE plots of DY and DO recipients are shown, with immune cell subpopulations color-coded. **D**–**L** Quantification of immune cell subsets of LPLs from DY (*n* = 9) and DO (*n* = 8) recipients was performed using standard gating: **D** Frequency of CD19^+^ B cells (*p* = 0.033) and **E** CD3^+^ T cells (*p* = 0.223) within CD45.1^+^ ileal LPLs. **F** Frequency of CD4^+^ T cells (*p* = 0.732) and **G** CD8^+^ T cells (*p* = 0.967) within CD3^+^ T cells of the ileal LP. **H** Representative dot plots and quantification of CD44^−^CD62L^+^ CD4^+^ T_N_ cells (*p* = 0.002), and **I** CD25^+^FoxP3^+^ T_reg_ cells (*p* = 0.040), within CD4^+^ T cells of the ileal LP. **J** Representative dot plots showing the gating strategy for CD44^−^CD62L^+^ T_N_ and CD44^+^CD62L^−^ T_EM_ CD8^+^ T cells. **K** Frequency of CD44^−^CD62L^+^ CD8^+^ T_N_ cells (*p* = 0.007) and **L** CD44^+^CD62L^−^ T_EM_ CD8^+^ T cells (*p* = 0.028) within CD8^+^ T cells of the ileal LP. Statistical significance was assessed using unpaired Student’s *t*-test (**C**–**G**, **J**–**K**). Data are presented as mean ± SD. Asterisks indicate statistical significance: *p < 0.05; **p < 0.01

To address these questions, young RAG1^−/−^ recipient mice were reconstituted with HSCs isolated from either young or old donors, generating mice with a young (DY) or old (DO) adaptive immune system in the circulating and splenic compartments, respectively (Fig. 3A). As RAG1^−/−^ mice lack endogenous B and T cells, all adaptive immune cells in these recipients are exclusively donor-derived. The phenotype of the resulting adaptive immune system is thus determined by the age of the transplanted HSCs, as previously demonstrated [29].

Consistent with earlier reports, overall donor cell engraftment (CD45.1^+^) in PB was lower in DO compared to DY mice (Suppl. Fig. 4A), reflecting the reduced repopulation potential of aged HSCs [53, 57, 58]. We next confirmed that DO recipients exhibited hallmark features of AAIR in PB when compared to DY mice (Suppl. Fig. 4A–H). Specifically, DO mice displayed reduced frequencies of CD19^+^ B cells (Suppl. Fig. 4B), lower proportions of CD4^+^ and CD8^+^ T_N_ cells (Suppl. Fig. 4E, F), and increased frequencies of memory T cell subsets among donor-derived CD45.1^+^ cells (Suppl. Fig. 4G, H).

We next examined whether donor-derived hematopoietic cells, as observed in PB, also populate the ileal LP. Quantitative analysis indicated that CD45.1^+^ donor cells constituted a substantial proportion—typically exceeding 50%—of total CD45^+^ cells in the ileal LP of both DY and DO recipients (Fig. 3B). Consistent with the reduced peripheral engraftment observed in DO mice, the proportion of donor-derived CD45.1^+^ cells was lower among ileal LP cells of DO in comparison to DY mice (Fig. 3B). Across individual mice, the extent of donor-derived cells in the ileal LP correlated strongly with that in PB (*r* = 0.9102), indicating a tight link between the level of systemic and mucosal engraftment (Suppl. Fig. 4I). These findings demonstrate that immune cells derived from transplanted HSCs can access and populate the gut mucosa. Hematopoiesis driven by HSCs located in the BM is thus also linked to the adaptive immune compartment of the ileal LP.

To test whether aged HSCs induce ileal AAIR, we compared immune landscapes between DY and DO recipients. Unsupervised viSNE projections on donor-derived CD45.1^+^ immune cells from the ileal LP showed overlapping maps but clear differences in specific compartments: DO samples displayed a lower representation of B cell clusters and a redistribution within the T cell compartment (Fig. 3C). Quantitative analysis showed that, while the frequencies of total CD3^+^ T cells and their major CD4^+^ and CD8^+^ T cell subsets were similar between DY and DO mice (Fig. 3E–G), more in-depth analysis revealed several hallmarks of AAIR in DO recipients. Both naïve CD4^+^ (Fig. 3H) and CD8^+^ (Fig. 3K) T cell frequencies were significantly reduced, while CD4^+^Foxp3^+^CD25^+^ T_reg_ cells (Fig. 3I) and CD8^+^ T_EM_ cells (Fig. 3L) showed increased frequency within LPLs of DO recipients. In addition, CD19^+^ B cell frequencies were reduced in DO mice (Fig. 3D). These changes in both the T and B cell compartments closely resembled those observed in naturally aged mice (Fig. 2G–K). The increase in T helper (T_h_) cell subsets such as T_h_17 (RORγt^+^) and T_h_2 (GATA3^+^) cells among CD3^+^CD4^+^ T cells, which we observed in the ileum of naturally aged mice (Suppl. Fig. 2I–K), is thought to result from cumulative antigen exposure [59, 60]. Their expansion may therefore be more closely linked to the duration of immune activation rather than aging per se. However, in the HSC transplantation model, T_h_17 cell frequencies were significantly increased in DO compared to DY recipients, and T_h_2 cells showed a trend toward higher frequency (Suppl. Fig. 4L–N). These findings suggest that immune-intrinsic HSCs driven aging also affects mucosal T_h_ cell composition, with a particularly pronounced effect on T_h_17 cells. In summary, these results show that HSC linked aging of the adaptive immune system leads to a local immune phenotype in the ileal LP that reflects key aspects of natural immune aging—especially within the T cell compartment.

In the myeloid compartment the frequency of Gr-1^+^/Mac-1^+^ innate immune cells remained comparable in the ileal LP of DO and DY mice, despite their increased abundance in PB of both O and DO mice (Suppl. Figs. 2C and 4 J, respectively). Notably, in comparison to lymphoid chimerism, a smaller fraction (<11%) of Gr-1^+^/Mac-1^+^ cells in the ileum were donor-derived (CD45.1^+^), while the vast majority originated from the recipient (CD45.2^+^) (Suppl. Fig. 4K). These findings suggest that that ileal myeloid populations are largely tissue resident and long-lived and resist replacement by BM-derived cells, at least under the mild transplant pre-conditioning regimen used in our experiments.

### AAIR affects vitamin B6 species in the gut and decreases the systemic availability of vitamin B6

**Fig. 4:**
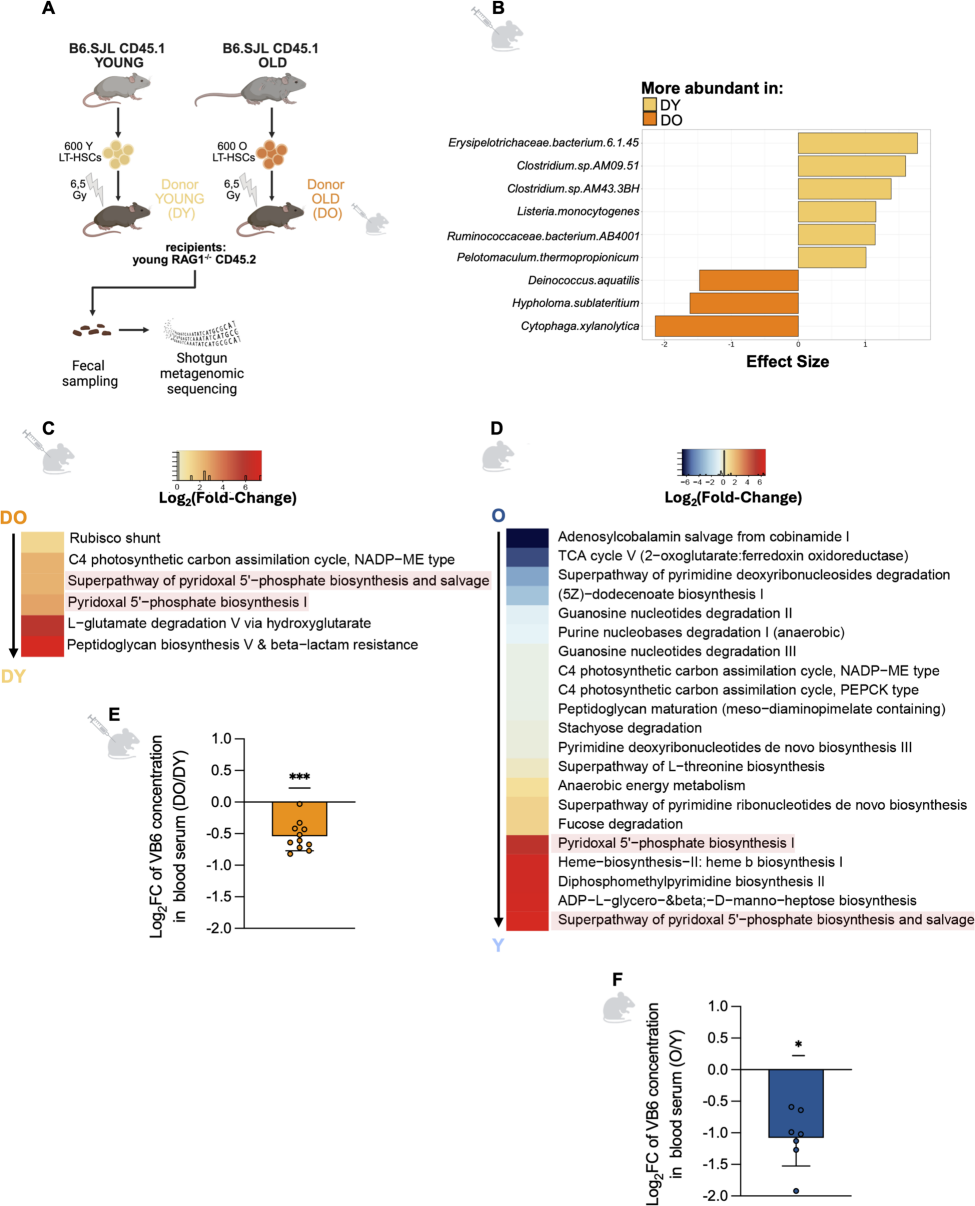
AAIR affects vitamin B6 species in the gut and decreases the systemic availability of vitamin B6. **A** Schematic of the experimental setup. Six hundred HSCs were isolated from young and old B6.SJL (CD45.1^+^) donors and subsequently transplanted into irradiated young RAG1^−/−^ (CD45.2^+^) recipients. Recipient mice were designated as DY (yellow) or DO (orange) based on the age of the respective HSC donors. Twenty weeks post-transplantation fecal samples were collected from DY and DO recipient mice. Microbial DNA was isolated and shotgun metagenomic sequencing was performed. **B** Effect size analysis of species-level microbial abundance based on fecal samples from DY (*n* = 15) and DO (*n* = 15) mice. The effect sizes of species significantly enriched in either DY (yellow) or DO (orange) mice are shown (*p *values for significant species are listed in Supplemental Table 4). Heatmaps showing differentially abundant microbial metabolic pathways based on pooled metagenomic gene content in **C** DY (*n* = 15) vs. DO (*n* = 15) recipient mice (*p *values are listed in Supplemental Table 6) and **D** in Y (*n* = 11) vs. O (*n* = 11) C57BL/6J mice (*p *values are listed in Supplemental Table 5). Color intensity represents relative pathway abundance: higher abundance in O or DO mice is shown in blue, lower abundance relative to Y or DY mice in red, with intermediate values represented along a continuous gradient. Log_2_ fold change (Log_2_FC) of vitamin B6 (VB6) concentrations in blood serum, measured by ELISA, comparing **E** DO vs. DY mice (*n* = 11 and *n* = 7, respectively; *p* < 0.001) and **F** O vs. Y (*n* = 4 both groups; *p* = 0.016). Y and DY groups were used as the reference (Log_2_FC = 0), respectively. Data are presented as individual Log_2_FC values, with each dot representing one biological replicate, calculated relative to the mean of the respective young reference group (DO/DY in **E**; O/Y in **F**); bars indicate mean ± SD. Effect sizes in **B** were derived using shrinkage linear discriminant analysis (SLDA) [61], and statistical significance was assessed using pairwise Wilcoxon rank-sum tests. Differential pathway abundance in **C** and **D** was assessed using pairwise Wilcoxon rank-sum tests on MetaCyc pathway abundances. Log2FC values in **E** and **F** were tested against the hypothetical value of 0 using a one-sample Wilcoxon signed-rank test. Data are presented as mean ± SD. Asterisks indicate statistical significance: *p < 0.05; ***p < 0.001

Hallmarks of AAIR in mucosal adaptive immune cells of the ileum are recapitulated in the HSC transplantation model in the absence of systemic aging (Fig. 3). We next investigated whether aging of the adaptive immune compartment in the gut contributes to aging-associated changes in the gut microbiota. To this end, we performed shotgun metagenomic sequencing on fecal samples collected 20 weeks post HSC transplantation from DY and DO mice (Fig. 4A). Three independent transplantation cohorts were analyzed, with DY and DO recipients transplanted on the same day and cohoused under standardized conditions within each cohort.

Despite variation in overall microbiota composition between independent experiments—likely reflecting maternal seeding/founder effects (distinct dams/litters), differences in microbial recolonization dynamics, housing conditions, or environmental exposure, and which are commonly encountered in microbiome studies [62], microbial community structure displayed a reproducible, while partially overlapping separation according to donor HSC age (DY vs. DO; Suppl. Fig. 5A–C). This result suggests that the age of the adaptive immune system might indeed confer an influence on microbial composition in the gut.

To further elucidate whether particular microbial taxa or patterns were robustly associated with immune age across cohorts, we pooled all DY and DO animals from the three cohorts (*n* = 15 per group) and performed a groupwise effect-size comparison. While each cohort contained more pronounced compositional changes, this pooled analysis focused on uncovering cross-cohort–consistent taxa that were differentially affected between DY and DO mice, despite baseline differences in cohort composition. This analysis revealed a small set of taxa differing significantly between DY and DO recipients (Fig. 4B), indicating their robust association with adaptive immune aging. However, there was no overlap between these species and those that significantly differed between naturally aged and young mice (Suppl. Fig. 1F). Together, these findings demonstrate that the age of the adaptive immune system can affect the composition of the gut microbiota. However, the small subset of consistently differing taxa, combined with the broader and distinctive changes observed within each cohort, suggests that the impact of adaptive immune aging is context-dependent. One possibility is that AAIR may not exert selective pressure primarily on specific taxa, but rather on broader microbial traits that are shared across different species, similar to the recently described microbiome-mediated clonal selection in hematopoiesis [63].

To test for traits, we analyzed the relative abundance of microbial metabolic pathways inferred from metagenomic gene content in DY and DO recipients. While such analyses estimate predicted metabolic potential rather than direct activity, pathway-level profiling from metagenomic data is a valid proxy for functional capacity, as metabolic gene abundance generally correlates with microbial ecological roles and with transcriptomic and metabolomic readouts [64–66]. Parallel pathway-level differential abundance analysis in Y and O C57BL/6J mice allowed us to assess whether similar alterations accompany natural systemic aging (Y vs. O; Fig. 4D) and adaptive immune aging (DY vs. DO; Fig. 4C). In Y versus O mice, we observed a broader pattern of age-associated pathway changes, including both increases and decreases in pathway abundance (Fig. 4D), whereas DO recipients predominantly exhibited decreases in pathway abundance relative to DY controls (Fig. 4C). Depleted pathways in DO mice included those involved in carbon metabolism (e.g., Rubisco shunt, C4 photosynthetic carbon assimilation), amino acid degradation (e.g., L-glutamate degradation V via hydroxyglutarate), and cell wall synthesis and resistance (e.g., peptidoglycan biosynthesis V, β-lactam resistance). Within this global screen, two VB6 biosynthesis and salvage pathways—the superpathway of pyridoxal 5′-phosphate biosynthesis and salvage (PWY0-845) and pyridoxal 5′-phosphate biosynthesis I (PYRIDOXSYN-PWY)—emerged as consistently reduced in both DO versus DY recipients and in naturally aged versus young C57BL/6J mice (Fig. 4C, D).

Motivated by this overlap, we performed targeted reanalysis of these two VB6 pathways using ALDEx2 with experimental batch modeled as a covariate (~ experiment + group) to account for batch associated variation. Both pathways showed substantially higher abundance in Y versus O and DY versus DO recipients (log_2_FCs: 6.6–8.6; Suppl. Fig. 5D, E). Although the two VB6 pathways showed large effect sizes and consistent directional changes across comparisons, neither remained significant after FDR correction across all the approximately 400 pathways tested, likely reflecting limited statistical power due to the small cohort sizes (*n* = 11–15; Suppl. Tables 7, 8). These results should therefore be interpreted as being consistent with, rather than definitively proving, reduced microbial VB6 biosynthetic capacity associated with natural aging and adaptive immune aging (Fig. 4C, D). Further validation in larger cohorts will be important to confirm the robustness of this association.

VB6 is a vital cofactor for enzymatic and signaling processes essential for amino acid metabolism, neurotransmitter synthesis, and immune regulation. In humans, lower systemic VB6 levels are observed in older adults [67] and associate with adverse outcomes including cardiovascular events, cognitive decline, increased mortality, and accelerated biological aging [68–70]. While diet provides the primary source, microbial biosynthesis in the gut substantially contributes to systemic VB6 availability [71]. Since metagenomic pathway abundance reflects the aggregate genetic capacity of VB6-producing microbes rather than direct metabolic activity, we measured serum VB6 levels to test whether reduced microbial VB6 biosynthetic potential correlates with lower host VB6 status. Serum VB6 concentrations were ~30% lower in DO recipients compared to DY counterparts, paralleling the ~50% reduction between naturally aged and young mice (Fig. 4E, F). These findings demonstrate that adaptive immune aging associates with both diminished presence of taxa with a VB6 biosynthetic capacity and reduced systemic VB6 levels, consistent with a model in which AAIR-driven microbiota changes may contribute to an age-associated decline in VB6.

## Discussion

Our data further support the concept of a bidirectional relationship between the immune system and the gut microbiome. Age-associated changes in the microbiota have been shown to influence immune function through altered metabolite production [72, 73], shifts in antigen exposure, or increased intestinal permeability [74, 75] that can promote low-grade inflammation [76, 77]. Our findings support the complementary view that aging of the immune system itself and specifically adaptive immune aging (AAIR) in the gut mucosa contribute to age-associated changes in the gut microbiota. While our experimental model might not capture all aspects of microbial aging, it will be useful to focus on aspects of immune intrinsic contributions to aging-associated changes of the microbiome and may inform immune-targeted microbiome modulating strategies to mitigate age-associated dysbiosis.

AAIR of the adaptive immune system is associated with a reduction in the abundance of microbial species harboring VB6 biosynthesis and salvage pathways in the gut and with decreased systemic availability of VB6. These findings are consistent with a scenario in which immune-mediated microbiome remodeling reduces the abundance of VB6-pathway–harboring taxa, thereby contributing to lower systemic VB6. While the observed reduction in microbial VB6 biosynthetic pathways is consistent with decreased microbial production, alternative mechanisms, such as increased VB6 demand by activated immune cells, changes in intestinal absorption, or systemic transport may also contribute to changes in levels of VB6. In addition, testing for functional VB6 deficiency using PLP-dependent metabolic readouts or assessing whether VB6 supplementation rescues downstream host phenotypes will be necessary to identify physiological consequences of the reduction in the level of VB6.

VB6 is essential for amino acid metabolism, neurotransmitter synthesis, and immune regulation [37]. These findings are potentially relevant to human aging, where comparable hallmarks of immune aging are well described in PB [78–82] and circulating VB6/PLP levels decline in many older adults [67, 83, 84]. Low VB6 levels in older adults have been associated with frailty [85, 86] and with biomarkers of accelerated biological aging, such as changes in epigenetic clocks [68]. In older individuals, low VB6 status has been mainly attributed to reduced dietary intake together with host metabolic factors [67, 87, 88]; our data raise the possibility that gut immune remodeling may also contribute low systemic levels of VB6 with age.

The observed shifts in ileal adaptive immune cell composition with age might result in functional consequences for host–microbiota interactions. In contrast, innate immune cell frequencies in the ileum remained stable and were predominantly of host origin, suggesting that these populations are long-lived, tissue-resident, and subject to minimal replacement by BM-derived hematopoiesis. This implies distinct turnover dynamics between the adaptive and innate immune compartments within the ileal mucosa.

One limitation of our data is that the underlying cellular and molecular mechanisms by which AAIR affects the abundance of VB6-producing species remains only partially addressed. The detailed mechanistic link between aged adaptive immune subsets and VB6-producing microbiota remains to be identified. As multiple adaptive cell compartments change in parallel with age, it is likely that aging-associated effects on the microbiome may arise from combined shifts in T- and B cell programs rather than from a single dominant subset.

In general, vulnerability of microbiota with a distinct metabolic pathway, such as VB6 biosynthesis and salvage, can be governed by the availability of limiting or toxic metabolites within or outside the microbiota. Very recent modeling of gut microbial interactions suggest that healthy states are characterized primarily by competition among taxa, whereas dysbiotic states feature reduced competition and increased cross-feeding [89]. While microbial effectors can inhibit VB6 biosynthesis pathways—as exemplified by pathogen-mediated targeting in other contexts [90]—it remains unclear whether aging-associated dysbiosis reduces VB6-proficient taxa primarily through reduced interspecies competition, increased cross-feeding (potentially delivering toxic metabolites), or their combination. Another possibility that connects changes in the immune system to changes in the microbiome might be specific immune reactions against a common immunological theme in VB6-producing species or any other taxa essential for supporting VB6-producing microbiota. These mechanisms are not mutually exclusive, and defining their relative contributions will require targeted functional experiments.

With respect to potential cellular mechanisms linking AAIR to VB6 pathway reductions, there is a possibility that reduced frequencies of T_N_ cells, lower B cell levels and decreased sIgA concentrations in the ileal lumen of aged mice (Suppl. Fig. 3A), along with increased proportions of effector and regulatory subsets, may reshape the mucosal environment in ways that selectively promote or restrict certain microbial taxa. Since mucosal IgA promotes niche protection and stable colonization of mutualistic taxa while limiting pathobionts [91–93], shifts in IgA quantity and/or specificity could change colonization fitness and thereby shift the community away from organisms carrying VB6 biosynthesis/salvage loci. In addition, the reduced responsiveness of CD4^+^ T cells from the aged ileal LP to stimulation (Suppl. Fig. 3B–E) may point to impaired immune control at the intestinal barrier. Such reduced immune responsiveness could permit the expansion of microbial taxa that are normally kept in check by intact mucosal immunity, thereby altering competitive dynamics within the community and indirectly disadvantaging taxa harboring VB6 biosynthesis/salvage pathways. Furthermore, T_h_17-associated programs are linked to epithelial integrity and inflammatory regulation [59], and changes in helper/regulatory balance may therefore influence microbial ecology through effects on epithelial function, mucus organization, and antimicrobial effector programs. While we currently cannot assign causality to specific cell subsets for their influence on VB6 taxa, targeted perturbation approaches, for example, adoptive transfer or depletion strategies to alter defined lymphocyte subsets, and interventions that perturb IgA induction, might identify which immune outputs contribute to the changes in frequency of VB6-associated taxa.

One candidate molecular mechanism that might link AAIR to reduced VB6 levels involves the ephrin type-B receptor 6 (EphB6). The EphB6 receptor tyrosine kinase is a member of the erythropoietin-producing hepatocyte (Eph) receptor family, which normally regulates cell-cell communication, cytoskeletal dynamics, and vesicle trafficking through interactions with ephrin ligands [94, 95]. A recent study by Li and colleagues demonstrated that whole-body knockout of *Ephb6* in mice alters the gut microbiota, and adoptive transfer experiments demonstrated that these changes in microbiota can lead to decreased systemic VB6 levels [96]. EphB6 is indeed highly expressed in T cells [97–99]. Expression analyses showed reduced *Ephb6* mRNA in PBMCs and a trend toward reduced expression in ileal LP CD4^+^ T cells in aged mice (Suppl. Fig. 6A, B). EphB6 appears to regulate, among others, cytoskeleton-dependent vesicle trafficking, docking, and exocytosis, as for example shown in adrenal gland chromaffin cells, in which it is responsible for exocytosis of catecholamine [100]. It is therefore a possibility that reduced levels of EphB6 may affect exocytosis, and the altered availability of that cargo might specifically affect VB6-producing microbiota. Further studies will be instrumental to clarify whether there is role of the EphB6 pathway in linking AAIR to host VB6 status.

Limitations of our experimental approach might need to be taken into account when interpreting our findings with respect to physiological aging. Immune aging and microbiome phenotypes can be sex-dependent; thus, our conclusions apply most directly to females and warrant future validation in males. The HSC transplantation model isolates hematopoietic age effects but introduces perturbations like irradiation, antibiotic exposure, and recolonization that may alter baseline microbiome composition and do not replicate the full aged tissue environment or lifelong antigen exposure shaping immune aging. Although HSCs originate from aged donors, immune reconstitution occurs within a young host over a compressed timeframe, lacking the gradual attrition, chronic antigenic stimulation, and cumulative epithelial–microbe interactions characteristic of natural aging [101]. In addition, laboratory housing limits environmental microbial exposure and ecological complexity [102], potentially restricting microbiome variability and responsiveness to immune aging. Physiological aging likely reflects additional environmental and tissue-intrinsic influences. Finally, the immune phenotyping was performed in the LP, whose organization differs across gut regions [47, 103], and microbiome profiling relied primarily on fecal samples, which mainly reflect distal colonic communities [104, 105] and may underrepresent region-specific immune–microbe interactions along the gut axis.

## Conclusion

In summary, our data reveal AAIR in the ileal LP that is recapitulated by transplantation of aged HSCs, establishing HSC aging as a contributor to aging-associated mucosal immune remodeling. This immune state is accompanied by altered gut microbiome structure and a consistent reduction in microbial VB6 biosynthesis/salvage pathway representation together with lower systemic VB6 availability. While the direct causal chain of immune remodeling, microbiome VB6 functional capacity, and host VB6 status remains to be established, these findings identify a gut mucosal axis through which hematopoietic immune aging may shape microbiome function and VB6 homeostasis during aging. By identifying the gut as a target organ of HSC-driven immune aging, our work provides rationale for future studies to determine the extent to which interventions that modulate immune aging and/or microbiome VB6 capacity might mitigate aging-associated changes in microbiome composition and VB6 availability.

## Supplementary Information

Supplementary Material 1.

## Data Availability

All data on which the conclusions of this study rely are publicly available. Metagenomics sequencing data are deposited in the NCBI Sequence Read Archive (SRA) under BioProject accession number PRJNA1320715. Original code is available via Zenodo under 10.5281/zenodo.15878856 Raw ileal LP CyTOF FCS files acquired on a Helios Mass Cytometer, together with associated metadata, have been deposited in Zenodo under 10.5281/zenodo.19653078. Additional data, including antibody lists and results of differential abundance analyses, are provided in the supplemental material.
