# Supplementary material for: Aging of the adaptive immune system affects the gut microbiome and systemic levels of vitamin B6
Source: Microbiome. 2026 Jun 5;14:163. doi: 10.1186/s40168-026-02428-3 (PMC13242121; doi:10.1186/s40168-026-02428-3)
Supplement: Supplementary file 2 — Supplementary Material 1. [file 40168_2026_2428_MOESM1_ESM.pdf]

Supplemental Figures
& Tables

Aging of the adaptive immune system affects the gut microbiome and systemic levels of vitamin B6

Selina Stahl, Hanna Widmaier, Vadim Sakk, Kodandaramireddy Nalapareddy, Ann-Kathrin Kissmann, Frank Rosenau, Medhanie A. Mulaw, David B. Haslam, Hartmut Geiger

Supplemental Figures

Suppl. Fig. 1: Adaptive immunity and age contribute to shape the composition of the gut microbiota.

(A–C) α-diversity of fecal microbiota was assessed using three complementary metrics: (A) Shannon index, (B) Simpson index, and (C) Chao1 richness estimator, in C57BL/6J (n = 11), RAG-HSC (n = 15), and RAG1$^{-/-}$ (n = 6) mice. **(D)** Firmicutes to Bacteroidetes (F:B) ratio in fecal samples from Y and O C57BL/6J mice (p = .15) **(E)** CLR–transformed species-level abundance of *Akkermansia muciniphila* in Y and O mice (p < .001). **(F)** Effect size analysis of species-level microbial abundance based on fecal samples from Y and O mice. Effect sizes of species significantly enriched in either Y (light blue) or O (dark blue) mice are shown. (*P*-values for significant species are listed in supplemental table 3). Panels (D-F) are based on the same groups of Y (n = 11) and O (n = 11) mice. Statistical significance was assessed using Wilcoxon rank-sum test for α-metrics (A-C), the F:B ratio (C) and for *A. muciniphila* abundance (D). . Effect sizes in (E) were derived using shrinkage linear discriminant analysis (SLDA), and statistical significance was assessed using pairwise Wilcoxon rank-sum tests. Data in (A-E) are presented as box plots showing the 0.25, 0.5 (median), and 0.75 percentiles, with whiskers indicating minimum and maximum values. In (C) bars represent the calculated effect size for each differentially abundant species. Asterisks indicate statistical significance: *p < .05; **p < .01; ***p < .001.

Suppl. Fig. 2: T cell-specific features of age-associated immune remodeling in blood are recapitulated in the aged ileal LP.

(A–G) Quantification of immune cell subsets within PB from Y (n = 8; 13–17 weeks old, median = 13) and O (n = 6; 97–98 weeks old, median = 97.5) C57BL/6J mice. **(A)** Frequency of CD19$^+$ B cells (p = .015), **(B)** CD3$^+$ T cells (p = .060), and **(C)** Gr-1$^+$Mac-1$^+$ myeloid cells (p = < .001) within CD45$^+$ PB cells. **(D)** Frequency of CD44$^-$CD62L$^+$ CD4$^+$ T_N cells (p < .001) within PB CD4$^+$ T cells. **(E)** Frequency of CD44$^-$CD62L$^+$ CD8$^+$ T_N cells (p < .001), **(F)** CD44$^+$CD62L$^+$ CD8$^+$ T_{CM} cells (p < .001), and **(G)** CD44$^+$CD62L$^-$ T_{EM} CD8$^+$ T cells (p < .001) within PB CD8$^+$ T cells. **(H–K)** Quantification of immune cell subsets within ileal LPLs from Y (n = 14; 12–18 months old, median = 13) and O (n = 12; 84–93 months old, median = 92) mice **(H)** Frequency of Gr-1$^+$Mac-1$^+$ myeloid cells (p = .317) within CD45$^+$ ileal LPLs. **(I)** Representative dot plots showing the gating strategy for RORγt$^+$ T_h17 cells and GATA3$^+$ T_h2 cells. **(J)** Frequency of RORγt$^+$ T_h17 cells (p = .049), and **(K)** GATA3$^+$ T_h2 cells (p = .001) within CD4$^+$ T cells of the ileal LP. Statistical significance was assessed using

unpaired Student's *t*-test (A–H, J–K). Data are presented as mean ± SD. Asterisks indicate statistical significance: **p* < .05; ***p* < .01; ****p* < .001.

Suppl. Fig. 3: Aging is associated with reduced mucosal sIgA levels and diminished cytokines responsiveness of ileal lamina propria CD4$^+$ T cells.

(A) Secretory IgA (sIgA) concentration in ileal contents from Y (n = 6) and O (n = 6) C57BL/6J mice, shown as Log_2FC with Y as the reference group (log_2[O/Y]). **(B,D)** Frequency of cytokine-positive cells among ileal LP CD4$^+$ T cells from Y(n =3) and O (n = 3) C57BL/6J mice after 5 h ex vivo stimulation with PMA/ionomycin in the presence of brefeldin A and monensin, followed by intracellular cytokine staining. Shown are **(B)** TNFα$^+$ CD4$^+$ T cells (*p* = .010) and **(C)** IL-2$^+$ CD4$^+$ T cells (*p* = .049). (**C, E**) Median cytokine signal intensity within the responding populations, shown as a proxy for per-cell cytokine abundance, for **(C)** TNFα (*p* = .090) and **(E)** IL-2 (*p* = .764). Data in (A) are presented as individual Log2FC values, with each dot representing one biological replicate, calculated relative to the mean of the young reference group; bars indicate mean ± SD. Data in (B–E) are shown for Y and O mice. Each dot represents one biological replicate (mouse); bars show mean ± SD. Statistical significance was assessed using unpaired Student's t-tests. Asterisks indicate statistical significance: **p* < .05; ***p* < .01.

Suppl. Fig. 4: Peripheral T cell remodeling induced by aged HSCs extends to the ileal LP of RAG1$^{-/-}$ mice.

(A–H) Quantification of immune cell subsets within PB from DY (n = 13) and DO (n = 15) recipients. **(A)** Donor contribution (CD45.1$^+$) to total CD45$^+$ PB population was assessed in DY and DO mice (*p* < .001). **(B)** Frequency of CD19$^+$ B cells (*p* = .008), **(C)** CD3$^+$ T cells (*p* = .826), and **(D)** Gr-1$^+$Mac-1$^+$ myeloid cells (*p* = .041) within donor-derived CD45.1$^+$ PB cells. **(E)** Frequency of CD44$^-$CD62L$^+$ CD4$^+$ T_N cells (*p* = .004) within donor-derived PB CD4$^+$ T cells. **(F)** Frequency of CD44$^-$CD62L$^+$ CD8$^+$ T_N cells (*p* = .005), **(G)** CD44$^+$CD62L$^+$ T_{CM} CD8$^+$ T cells (*p* = .049), and **(H)** CD44$^+$CD62L$^-$ T_{EM} CD8$^+$ T cells (*p* = .024) within donor-derived PB CD8$^+$ T cells. **(I)** Donor contribution (CD45.1$^+$) to total CD45$^+$ cells in PB and ileal LPLs was compared within individual mice. Pearson correlation analysis was performed; the solid line indicates the regression fit, dotted lines represent the 95% confidence interval, and the correlation coefficient (r = 0.9102) is shown. **(J–N)** Quantification of immune cell subsets within ileal LPLs from DY (J,K: n = 6; L-N: n = 9) and DO (J,K: n = 6; L-N: n = 8) recipients **(J)** Frequency of Gr-1$^+$Mac-1$^+$ myeloid cells within donor-derived CD45.1$^+$ ileal LPLs. **(K)** Representative dot plots and quantification of CD45.1$^+$ donor-derived Gr-1$^+$Mac-1$^+$ myeloid cells within total ileal LP Gr-1$^+$Mac-1$^+$ myeloid cells (CD45.1$^+$ and CD45.2$^+$). **(L)**

Representative dot plots showing the gating strategy for RORγt$^+$ T_h17 cells and GATA3$^+$ T_h2 cells. **(M)** Frequency of RORγt$^+$ T_h17 cells (p = .034), and **(N)** GATA3$^+$ T_h2 cells (p = .358) within donor-derived CD4$^+$ T cells of the ileal LP. Statistical significance was assessed using unpaired Student's *t*-test (A–J, L–M) and Pearson correlation analysis (I). Data are presented as mean ± SD. Asterisks indicate statistical significance: *p < .05; **p < .01; ***p < .001.

Suppl. Fig. 5: AAIR induces distinct shifts in the gut microbial composition and pathway abundance.
(A-C) PCA of CLR–transformed species-level microbial abundances, showing distinct clustering by mouse group. Samples are color-coded by group: DY (yellow) and DO (orange). Each panel represents one independent experiment in which DY and DO mice, transplanted on the same day, clustered separately (*P*-values are listed in supplemental table 4). Ellipses indicate 95% confidence regions for each group. **(A)** PCA plot of experiment 1 (DY: n = 6; DO: n = 5), **(B)** experiment 2 (DY: n = 4; DO: n = 4), and **(C)** experiment 3 (DY: n = 5; DO: n = 6). **(D, E)** Log_2FC for two microbial VB6 biosynthesis/salvage pathways – PYRIDOXSYN-PWY (pyridoxal 5′-phosphate biosynthesis I) and PWY0-845 (superpathway of pyridoxal 5′-phosphate biosynthesis and salvage) – are shown for each comparison. **(D)** Y versus O and **(E)** DY versus DO, with O and DO used as the respective reference groups; positive values therefore indicate higher pathway abundance in Y or DY mice (*P*-values are listed in supplemental table 7 and 8, respectively). Statistical significance was assessed using MRPP for PCA clustering (A-C). Pathway differential abundance in **(D,E)** was assessed using an ALDEx2 generalized linear model including sequencing run as a covariate (model formula: ~ Experiment + Group).

Suppl. Fig. 6: Reduced *Ephb6* mRNA expression in old immune cells.
(A) Relative *Ephb6* mRNA expression in peripheral blood mononuclear cells (PBMCs) from young (Y; n = 8) and old (O; n = 6) C57BL/6J mice (p = .002). **(B)** Relative *Ephb6* expression in CD4$^+$ ileal lamina propria lymphocytes (LPLs) from Y (n = 3) and O (n = 3) mice (p = .133). *Ephb6* transcript levels were quantified by quantitative polymerase chain reaction (qPCR), normalized to glyceraldehyde 3-phosphate dehydrogenase (*Gapdh*), and expressed as $2^{-\Delta\Delta Ct}$ referenced to the mean Y group (dashed line indicates the reference level, $2^{-\Delta\Delta Ct} = 1$). Each dot represents one biological replicate (mouse); bars show mean ± SD. Statistical significance was assessed using an unpaired Welch's t-test on ΔCt values. Asterisks indicate statistical significance: **p < .01.

Supplemental Tables

Suppl. Table 1: Antibodies used for flow cytometry analyses.
The table lists the target antigen, fluorochrome conjugate, clone name, dilution, supplier, and catalog number for each antibody used in flow cytometric experiments.

Suppl. Table 2: Antibodies used for CyTOF analyses.
The table lists the target antigen, metal tag, clone name, dilution, supplier, and catalog number for all antibodies used in CyTOF experiments.

Suppl. Table 3: Differential species abundance in fecal samples of Y and O C57BL/6J mice.
Statistical significance was assessed using the Wilcoxon rank-sum test followed by false discovery rate (FDR) correction. The table includes unadjusted *p*-values and FDR-adjusted *q*-values for each microbial species. Listed are species showing nominally significant differences in abundance, including those that did not remain significant after FDR correction. A subset of these species with the highest effect sizes, as determined by SLDA, is visualized in Suppl. Fig. 1F.

Suppl. Table 4: Differential species abundance in fecal samples of DY and DO mice.
Statistical comparisons were performed using the Wilcoxon rank-sum test followed by FDR correction. The table includes unadjusted *p*-values and FDR-adjusted *q*-values for each microbial species. While no species reached statistical significance after FDR correction (FDR < 0.1), species with the lowest unadjusted *p*-values ($p < 0.05$) are listed here. A subset of these species, selected based on effect size, is visualized in Fig. 4B**.**

Suppl. Table 5: Differential microbial pathway abundance in fecal samples of Y and O C57BL/6J mice.
Statistical significance was assessed using the Wilcoxon rank-sum test followed by FDR correction. The table includes pathway-level abundance data (mean values per group), absolute differences, fold change (FC), log-ratio, unadjusted *p*-values, and FDR-adjusted *q*-values. Pathways showing nominal differences in abundance are listed, and those meeting the FDR threshold are indicated accordingly. All pathways shown in Fig. 4D are included in this table.

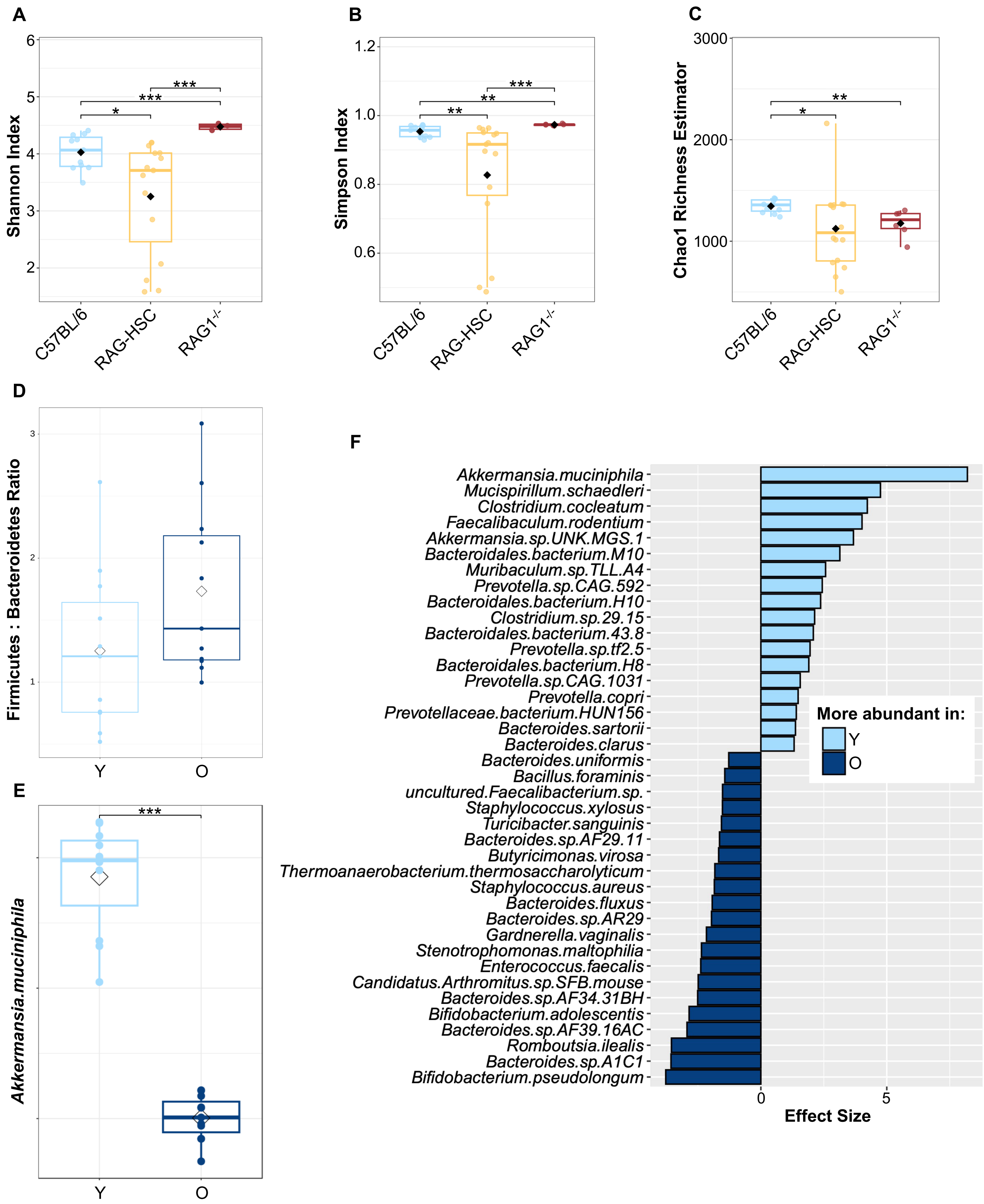

Supplementary Figure 1 Stahl et al.

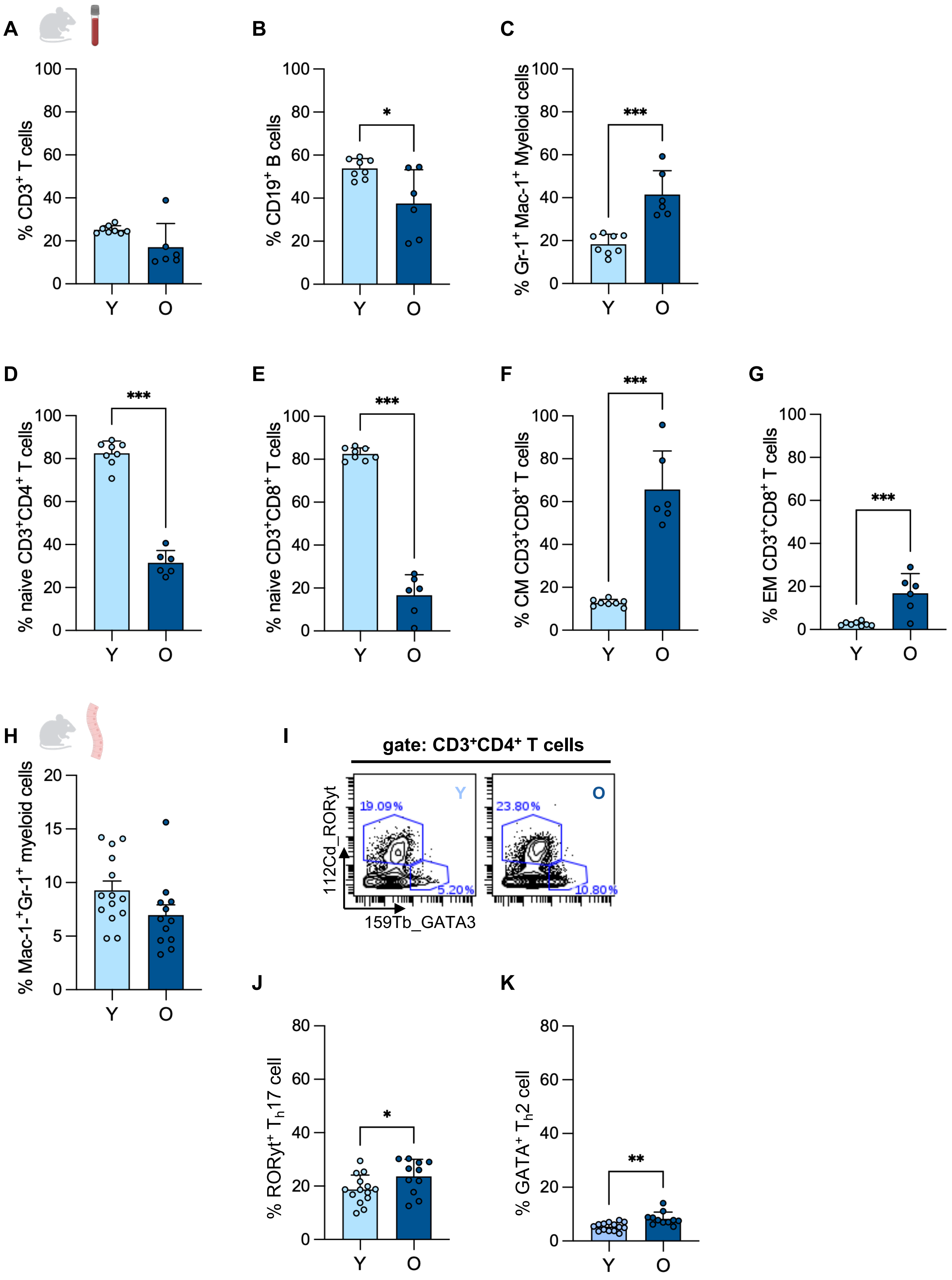

Supplementary Figure 2 Stahl et al.

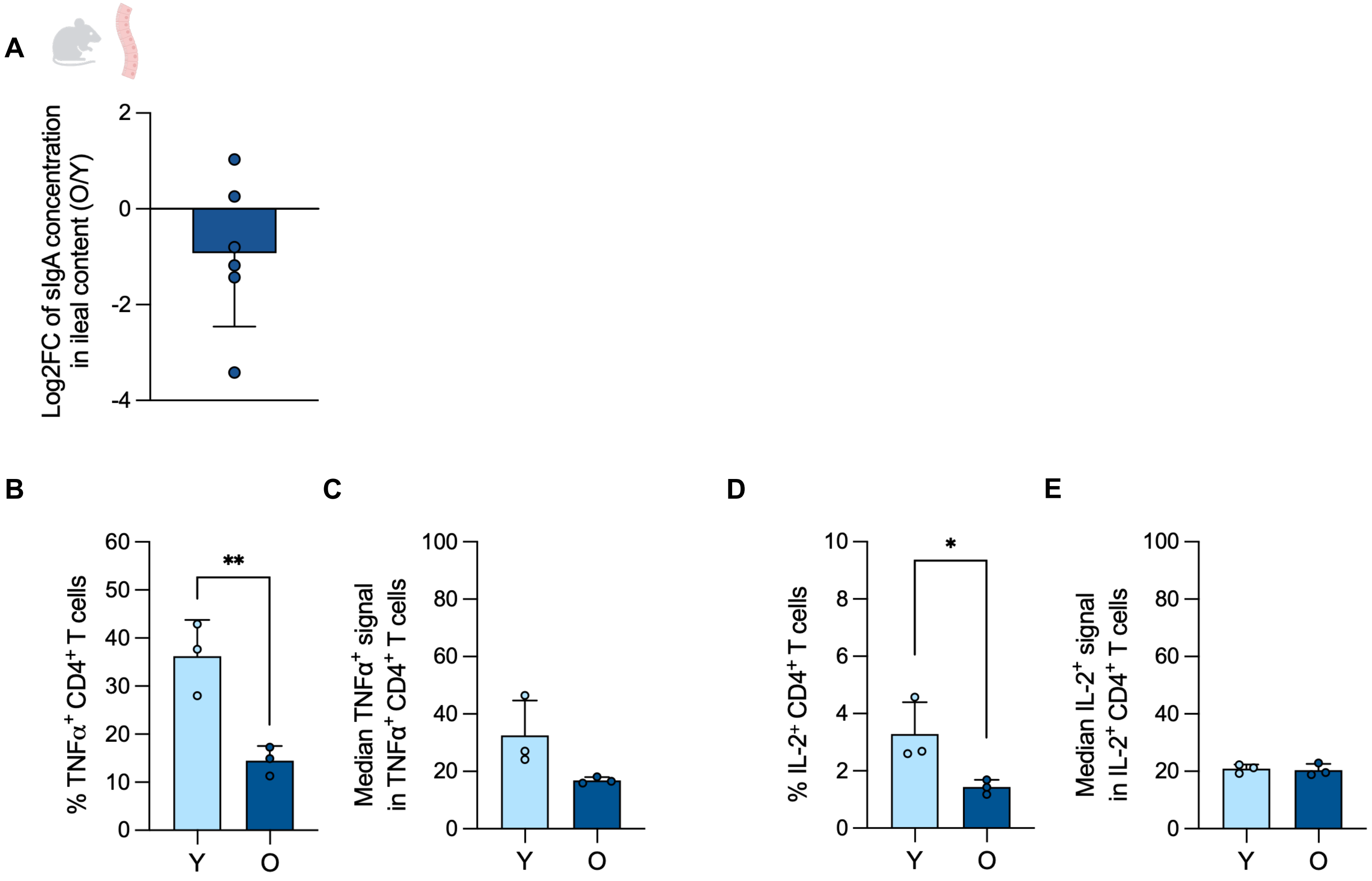

Supplementary Figure 3 Stahl et al.

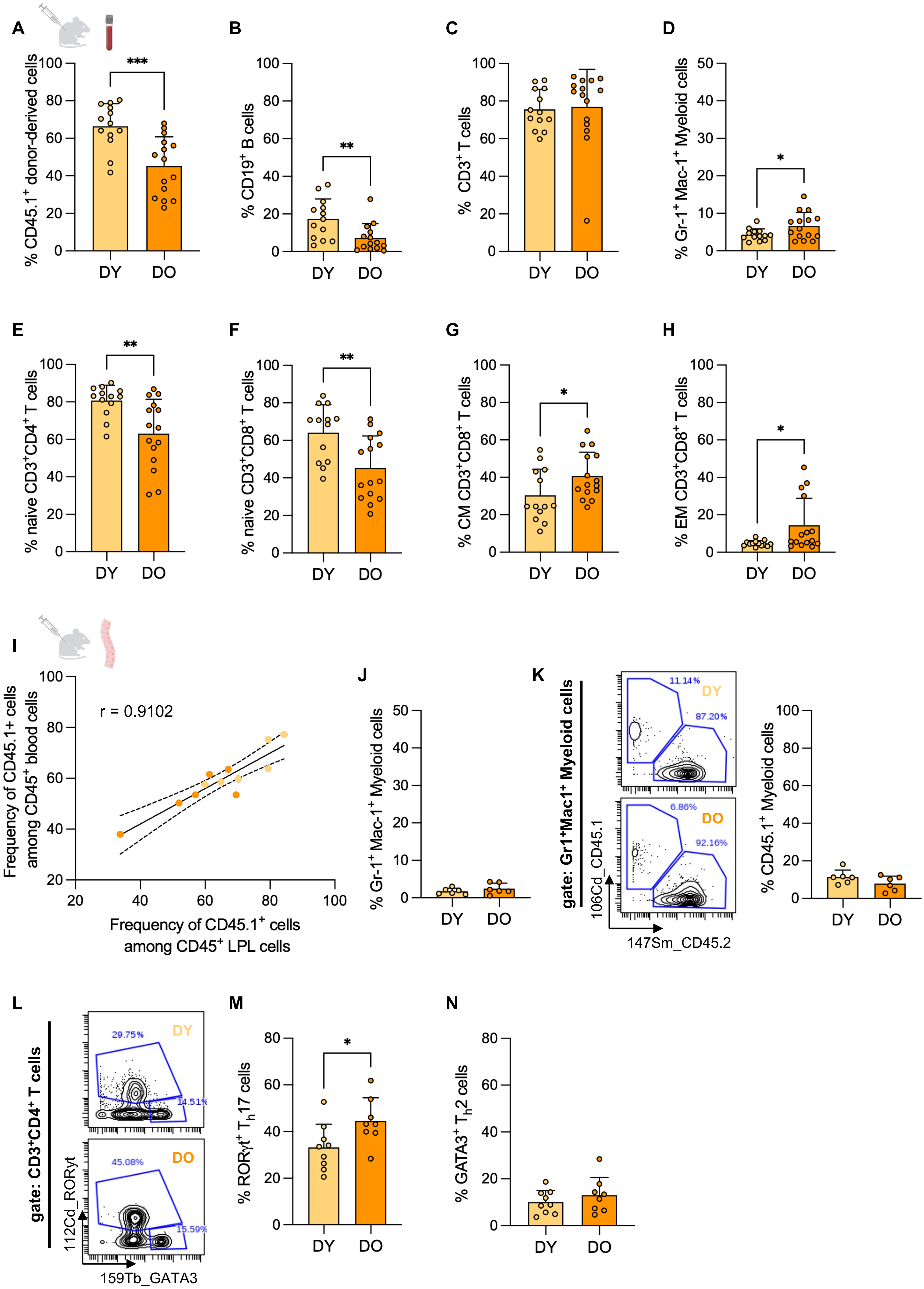

Supplementary Figure 4 Stahl et al.

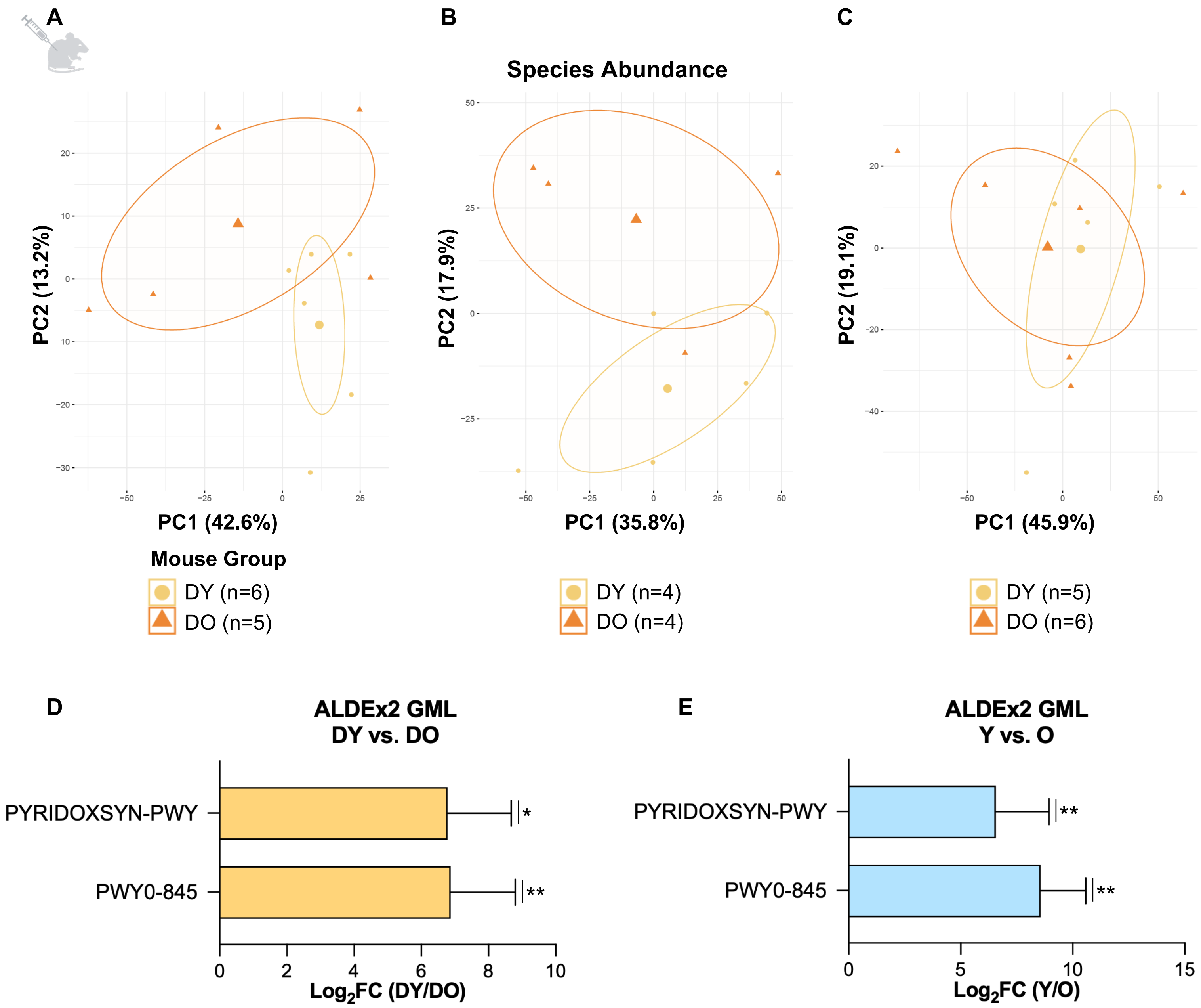

Supplementary Figure 5 Stahl et al.

Suppl. Table 6: Differential microbial pathway abundance in fecal samples of DY and DO mice.

Statistical significance was assessed using the Wilcoxon rank-sum test followed by FDR correction. The table includes pathway-level abundance data (mean values per group), absolute differences, FC, log-ratio, unadjusted p-values, and FDR-adjusted q-values. Pathways showing nominal differences in abundance are listed, and those meeting the FDR threshold are indicated accordingly. All pathways shown in Fig. 4C are included in this table.

Suppl. Table 7: Targeted ALDEx2 analysis of microbial VB6 biosynthesis/salvage pathways in Y and O mice.

Pathway differential abundance between Y and old O C57BL/6J mice was assessed using a targeted ALDEx2 generalized linear model including sequencing run as a covariate (model formula: ~ Experiment + Group). The table includes the estimated $\log_2$ fold change (Log2FC), standard error (SE), *t*-value, unadjusted *p*-value, and FDR-adjusted *q*-value for each pathway. O mice were used as the reference group; thus, positive estimates indicate higher pathway abundance in Y mice. The two vitamin B6-associated pathways shown in Suppl. Fig. 5D are included.

Suppl. Table 8: Targeted ALDEx2 analysis of microbial VB6 biosynthesis/salvage pathways in DY and DO mice.

Pathway differential abundance between donor young (DY) and donor old (DO) mice was assessed using a targeted ALDEx2 generalized linear model including sequencing run as a covariate (model formula: ~ Experiment + Group). The table includes the estimated Log_2FC, SE, *t*-value, unadjusted *p*-value, and FDR-adjusted *q* value for each pathway. DO mice were used as the reference group; thus, positive estimates indicate higher pathway abundance in DY mice. The two vitamin B6-associated pathways shown in Suppl. Fig. 5E are included.

Suppl. Table 1: Antibodies used for flow cytometry analyses.

Antibody	Dilution	Supplier	Identifier
anti-Ly-6A/E (Sca-1) Pe-Cy7 (clone: D7)	1:200	eBiosciences	Cat# 25-5981-82
anti-CD117(ckit) APC (clone: ACK2)	1:50	eBiosciences	Cat# 47-1171-82
anti-CD135 (Flt3) PE (clone: A2F10)	1:100	eBiosciences	Cat# 12-1351-83
anti-CD34 FITC (clone: RAM34)	1:50	eBiosciences	Cat# 11-0341-85
Streptavidin eFluor 450	1:100	eBiosciences	Cat# 48-4317-82
anit-CD3 PE-Cy7 (clone: 145-2C11)	1:300	eBioscience	Cat# 25-0031-82
anti-CD8 Pacific Blue (clone: 53-6.7)	1:100	BD Pharmingen	Cat# 558106
anti-CD4 APC (clone: RAM4-5)	1:200	eBioscience	Cat# 17-0042-83
anti-CD44 FITC (clone: IM7)	1:200	BD Pharmingen	Cat# 553133
anti-CD62L PerCPCy5.5 (clone: MEL-14)	1:200	eBioscience	Cat# 45-0621-82
anti-CD49d PE (clone: R1-2)	1:300	BioLegend	Cat# 103607
anti-Gr-1 eFluor450 (clone: RB6-8C5)	1:100	ebioscience	Cat# 48-5931-82
anti-MAC-1 AlexaFluor700 (clone: M1/70)	1:100	ebioscience	Cat# 56-0112-82
anti-CD19 APC (clone: eBio1D3)	1:200	ebioscience	Cat# 17-0193-82
anti-CD45.1 PE (clone: A20)	1:300	eBioscience	Cat# 12-0453-82
anti-CD45.2 APC-Vio770 (clone: 104-2)	1:200	Miltenyi Biotec	Cat# 130-119-128

Suppl. Table 2: Antibodies used for CyTOF analyses.

Antibody	Dilution	Supplier	Identifier
anti-CD21/CD35 110Cd (clone: eBio8D9)	1:50	eBioscience	Cat# 14-0211-81
anti-RORyt 112Cd (clone: Q31-378)	1:100	BD Pharmingen	Cat# 562663
anti-CD45.1 106Cd (clone: A20)	1:100	eBioscience	Cat# 14-0453-82
anti-Ly6G/C (Gr-1) 141Pr (clone: RB6-8C5)	1:100	Standard BioTools	Cat# 201306
anti-CD11c 142Nd (clone: N418)	1:100	Standard BioTools	Cat# 201306
anti-CD69 145Nd (clone: H1.2F3)	1:100	Standard BioTools	Cat# 201306
anti-CD45.2 147Sm (clone: 104)	1:100	eBioscience	Cat# 14-0454-82
anti-CD11b (Mac-1) 148Nd (clone: M1/70)	1:100	Standard BioTools	Cat# 201306
anti-CD19 149Sm (clone: 6D5)	1:100	Standard BioTools	Cat# 201306
anti-CD25 (IL-2R) 151Eu (clone: 3C7)	1:100	Standard BioTools	Cat# 201306
anti-CD3e 152Sm (clone: 145-2C11)	1:100	Standard BioTools	Cat# 201306
anti-CD23 153Eu (clone: B3B4)	1:100	BioLegend	Cat# 101625
anti-TER-119 154Sm (clone: TER-119)	1:100	Standard BioTools	Cat# 201306
anti-CD90.2/Thy-1.2 156Gd (clone: 30-H12)	1:500	BioLegend	Cat# 105333
anti-GATA3159Tb (clone: TWAJ)	1:100	eBioscience	Cat# 14-9966-82
anti-CD62L 160Gd (clone: MEL-14)	1:100	Standard BioTools	Cat# 201306
anti-TCRgd 163Dy (clone: GL3)	1:100	eBioscience	Cat# 14-5711-82
anti-FoxP3164Dy (clone: 3G3)	1:100	Invitrogen	Cat# MA5-44011
anti-CD8a 168Er (clone: 53-6.7)	1:100	Standard BioTools	Cat# 201306
anti-TCRb 169Tm (clone: H57-597)	1:100	Standard BioTools	Cat# 201306
anti-CD161 (NK1.1) 170Er (clone: PK136)	1:100	Standard BioTools	Cat# 201306
anti-CD44 171Yb (clone: IM7)	1:200	Standard BioTools	Cat# 201306
anti-CD4 172Yb (clone: RM4-5)	1:100	Standard BioTools	Cat# 201306

anti-CD45R (B220) 176Yb (clone: RA3-6B2)	1:100	Standard BioTools	Cat# 201306
anti-TNF-α 162Dy (clone: MP6-XT22)	1:100	Standard BioTools	Cat# 201310
anti-IL-2 144Nd JES6-5H4	1:100	Standard BioTools	Cat# 201310
Iridium 191Ir	1:100	Standard BioTools	Cat# 201306
Iridium 193Ir	1:100	Standard BioTools	Cat# 201306

Suppl. Table 3: Differential species abundance in fecal samples of Y and O C57BL/6J mice.

Species	*p*_Unadjusted	FDR
Clostridium.sp.29.15	0,0027	0,0384
Akkermansia.sp.54.46	0,0028	0,0384
Akkermansia.sp.CAG.344	0,0028	0,0384
Akkermansia.sp.UNK.MGS.1	0,0028	0,0384
Burkholderiales.bacterium.1.1.47	0,0028	0,0384
Parasutterella.excrementihominis	0,0028	0,0384
Lysinibacillus.chungkukjangi	0,0096	0,0494
Akkermansia.muciniphila	0,0022	0,0384
Faecalibaculum.rodentium	0,0022	0,0384
Muribaculum.sp.TLL.A4	0,0022	0,0384
Bacteroidales.bacterium.43.8	0,0067	0,0453
Clostridium.sp.AM43.3BH	0,0438	0,1127
Mucispirillum.schaedleri	0,0411	0,1084
Prevotella.sp.CAG.1031	0,0022	0,0384
Pleurotus.salmoneostramineus	0,0367	0,1028
Prevotella.sp.CAG.1185	0,0022	0,0384
Prevotella.sp.CAG.592	0,0050	0,0384
Prevotella.sp.KCOM.3155	0,0022	0,0384
Clostridium.cocleatum	0,0022	0,0384
Prevotella.bryantii	0,0050	0,0384
Bacteroidales.bacterium.H7	0,0022	0,0384
Bacteroidales.bacterium.H9	0,0022	0,0384
Bacteroides.sp.CF01.10NS	0,0100	0,0497
Bilophila.wadsworthia	0,0303	0,0883
Prevotella.sp.CAG.1124	0,0022	0,0384
Burkholderiales.bacterium	0,0050	0,0384
Prevotella.sp.CAG.1058	0,0050	0,0384
Bacteroidales.bacterium.H3	0,0022	0,0384
Prevotella.oris	0,0050	0,0384
Prevotella.sp.KH2C16	0,0022	0,0384
Clostridium.grantii	0,0260	0,0804
Coprococcus.sp.TF11.13	0,0043	0,0384
Clostridium.sp.OM05.9	0,0022	0,0384
Prevotella.sp.CAG.255	0,0022	0,0384
Prevotella.enoeca	0,0050	0,0384
Erysipelatoclostridium.ramosum	0,0450	0,1127
Prevotella.buccalis	0,0050	0,0384
Bacteroides.sp.43.108	0,0050	0,0384
Amanita.inopinata	0,0126	0,0554
Parabacteroides.johnsonii	0,0050	0,0384
Prevotella.fusca	0,0049	0,0384
Parabacteroides.sp.SN4	0,0050	0,0384
Bacteroidales.bacterium.M10	0,0087	0,0461
Prevotella.sp.tf2.5	0,0200	0,0709
Bacteroides.sp.CAG.754	0,0152	0,0606
Desulfovibrionaceae.bacterium	0,0043	0,0384
Prevotella.sp.CAG.1092	0,0049	0,0384
Prevotella.sp.CAG.1320	0,0022	0,0384
Bacteroides.sp.3.1.23	0,0370	0,1028
Prevotella.marshii	0,0050	0,0384
Clostridium.sp.AM34.9AC	0,0087	0,0461
Lactobacillus.animalis	0,0200	0,0709
Bacteroides.sp.AM32.11AC	0,0370	0,1028

Blautia.sp.AF19.34	0,0159	0,0606
Prevotella.maculosa	0,0050	0,0384
Prevotella.sp.CAG.520	0,0050	0,0384
Campylobacter.fetus	0,0260	0,0804
Bacteroides.sp.D2	0,0152	0,0606
Prevotella.buccae	0,0043	0,0384
Prevotella.shahii	0,0049	0,0384
Prevotella.intermedia	0,0022	0,0384
Bacteroides.sp.AM16.13	0,0303	0,0883
Prevotella.sp.Marseille.P4334	0,0100	0,0497
Prevotella.copri	0,0022	0,0384
Prevotella.sp.Marseille.P4119	0,0043	0,0384
Lachnospiraceae.bacterium.COE1	0,0411	0,1084
Bacteroides.sp.3.1.13	0,0152	0,0606
Prevotellaceae.bacterium.HUN156	0,0103	0,0500
Bacteroides.sp.CAG.530	0,0022	0,0384
Bacteroidales.bacterium.M3	0,0022	0,0384
Prevotella.sp.CAG.474	0,0062	0,0433
Prevotella.albensis	0,0050	0,0384
uncultured.Prevotella.sp.	0,0022	0,0384
Bacteroidales.bacterium.M2	0,0022	0,0384
Prevotella.melaninogenica	0,0050	0,0384
Bacteroides.finegoldii	0,0152	0,0606
Bacteroides.faecis	0,0087	0,0461
Bacteroidales.bacterium.Phil12	0,0087	0,0461
Prevotella.brevis	0,0129	0,0554
Prevotella.paludivivens	0,0043	0,0384
Ignavibacteriales.bacterium.CG.4.9.14.3.um.filter.34.10	0,0043	0,0384
Prevotella.denticola	0,0081	0,0461
Bacteroides.sp.AM16.15	0,0022	0,0384
Bacteroidales.bacterium.GP2	0,0050	0,0384
Prevotella.ihumii	0,0081	0,0461
Prevotella.sp.DNF00663	0,0022	0,0384
Bacteroides.caccae.CAG.21	0,0409	0,1084
Prevotella.amnii	0,0064	0,0433
Flavobacterium.anhuiense	0,0064	0,0433
Bacteroides.ovatus	0,0022	0,0384
Bacteroides.caccae	0,0087	0,0461
Prevotella.saccharolytica	0,0260	0,0804
Prevotella.sp.MA2016	0,0152	0,0606
Prevotella.baroniae	0,0064	0,0433
Prevotella.bivia	0,0050	0,0384
Prevotella.colorans	0,0081	0,0461
Rubellimicrobium.thermophilum	0,0129	0,0554
Bacteroidales.bacterium.H5	0,0022	0,0384
Bacteroidales.bacterium.KHT7	0,0126	0,0554
Prevotella.corporis	0,0078	0,0461
Bacteroides.xylanisolvens	0,0260	0,0804
Prevotella.sp.ne3005	0,0242	0,0804
Prevotella.pallens	0,0049	0,0384
Prevotella.stercorea	0,0050	0,0384
Bacteroides.sp.OF03.11BH	0,0159	0,0606
Prevotella.sp.TF12.30	0,0129	0,0554
Prevotella.veroralis	0,0022	0,0384
Prevotella.timonensis	0,0050	0,0384
Prevotella.sp.CAG.924	0,0022	0,0384
Bacteroidales.bacterium.KA00344	0,0161	0,0606
Bacteroidales.bacterium.H8	0,0196	0,0709

Prevotella.ruminicola	0,0200	0,0709
Prevotella.disiens	0,0129	0,0554
Candidatus.Homeothermus.arabinoxylanisolvens	0,0022	0,0384
Prevotella.oryzae	0,0260	0,0804
Bacteroides.sp.Marseille.P3684	0,0064	0,0433
uncultured.Prevotellaceae.bacterium	0,0022	0,0384
Bacteroidales.bacterium.K1	0,0043	0,0384
Paeniclostridium.sordellii	0,0367	0,1028
Parabacteroides.goldsteinii	0,0043	0,0384
Prevotella.multiformis	0,0200	0,0709
bacterium.P201	0,0043	0,0384
Bacteroidales.bacterium.H1	0,0050	0,0384
Butyricimonas.faecihominis	0,0078	0,0461
Prevotella.multisaccharivorax	0,0126	0,0554
Cronobacter.dublinensis	0,0087	0,0461
Bacteroides.clarus	0,0043	0,0384
Bacteroides.pyogenes	0,0087	0,0461
Prevotellaceae.bacterium	0,0158	0,0606
Bacteroidales.bacterium.M5	0,0450	0,1127
Mycolicibacterium.chubuense	0,0099	0,0497
Bacteroides.sartorii	0,0087	0,0461
Tricholoma.matsutake	0,0159	0,0606
Prevotella.bergensis	0,0260	0,0804
Bacteroidales.bacterium.M9	0,0022	0,0384
Bacteroides.caecimuris	0,0260	0,0804
Pandoraea.thiooxydans	0,0370	0,1028
Bacteroidales.bacterium.H6	0,0152	0,0606
Butyricimonas.synergistica	0,0087	0,0461
Bacteroidales.bacterium.GP3	0,0022	0,0384
Bacteroidales.bacterium	0,0260	0,0804
Parabacteroides.sp.D13	0,0450	0,1127
Porphyromonas.cangingivalis	0,0357	0,1025
Bacteroides.sp.3.1.33FAA	0,0129	0,0554
Trichosporon.faecale	0,0247	0,0804
Petrimonas.mucosa	0,0247	0,0804
Achromobacter.denitrificans	0,0161	0,0606
Dysgonomonas.sp.HGC4	0,0303	0,0883
Bacteroidales.bacterium.GP4	0,0260	0,0804
Bacteroidales.bacterium.52.46	0,0260	0,0804
Coprobacter.sp.	0,0367	0,1028
Clostridium.beijerinckii	0,0411	0,1084
Streptococcus.canis	0,0431	0,1127
Bacteroidales.bacterium.GP1	0,0411	0,1084
Bacteroidales.bacterium.H10	0,0260	0,0804
Parabacteroides.sp.AM08.6	0,0301	0,0883
Holdemania.sp.Marseille.P2844	0,0438	0,1127
Hespellia.stercorisuis	0,0411	0,1084
Clostridium.symbiosum	0,0087	0,0461
Clostridiales.bacterium.Firm.11	0,0446	0,1127
Mesorhizobium.sp.	0,0354	0,1021
Clostridium.citroniae	0,0370	0,1028
Blautia.wexlerae	0,0411	0,1084
Candidatus.Rokubacteria.bacterium	0,0303	0,0883
Clostridium.sp.AF23.8	0,0303	0,0883
Raoultibacter.timonensis	0,0303	0,0883
Lachnospiraceae.bacterium.3.2	0,0152	0,0606
Clostridium.sp.AF18.27	0,0411	0,1084
Butyrivibrio.sp.LB2008	0,0077	0,0461

Lachnospiraceae.bacterium.2.1.46FAA	0,0450	0,1127
uncultured.Erysipelotrichaceae.bacterium	0,0446	0,1127
Chloroflexi.bacterium	0,0022	0,0384
Bacteroidetes.bacterium	0,0446	0,1127
Roseburia.sp.CAG.309	0,0411	0,1084
Firmicutes.bacterium.CAG.110	0,0450	0,1127
Anaerostipes.sp.AF04.45	0,0370	0,1028
Ruminococcaceae.bacterium.cv2	0,0411	0,1084
Ruminococcus.gauvreauii	0,0411	0,1084
Butyrivibrio.sp.INlla21	0,0303	0,0883
Clostridium.lavalense	0,0411	0,1084
Halanaerobium.saccharolyticum	0,0446	0,1127
Clostridium.butyricum	0,0245	0,0804
Clostridium.sp.CAG.448	0,0438	0,1127
Lachnospiraceae.bacterium.MC2017	0,0446	0,1127
Anaerostipes.caccae	0,0161	0,0606
uncultured.Ruminococcus.sp.	0,0260	0,0804
Hungatella.hathewayi	0,0411	0,1084
Firmicutes.bacterium.CAG.646	0,0260	0,0804
Clostridiaceae.bacterium.14S0207	0,0438	0,1127
Clostridium.sp.AM33.3	0,0260	0,0804
Anaerobacterium.chartisolvens	0,0200	0,0709
Rhodopseudomonas.palustris	0,0367	0,1028
Eisenbergiella.tayi	0,0152	0,0606
Ruminococcus.albus	0,0200	0,0709
Phormidesmis.priestleyi	0,0129	0,0554
Ruminococcaceae.bacterium.Marseille.P2935	0,0152	0,0606
Clostridium.sp.D5	0,0411	0,1084
Ruminococcus.lactaris	0,0152	0,0606
Dorea.longicatena	0,0043	0,0384
Ruminococcus.sp.AF41.9	0,0367	0,1028
Dorea.sp.D27	0,0411	0,1084
Anaerotignum.propionicum	0,0260	0,0804
Uromyces.viciae.fabae	0,0367	0,1028
Eubacterium.plexicaudatum	0,0022	0,0384
Lachnospiraceae.bacterium.NK4A144	0,0260	0,0804
Pseudoclostridium.thermosuccinogenes	0,0411	0,1084
Arabia.massiliensis	0,0198	0,0709
uncultured.Roseburia.sp.	0,0022	0,0384
Clostridium.sp.Marseille.P2415	0,0450	0,1127
Blautia.sp.CAG.257	0,0043	0,0384
Pedobacter.sp.	0,0101	0,0497
Acetonema.longum	0,0298	0,0883
uncultured.Blautia.sp.	0,0411	0,1084
Eubacterium.oxidoreducens	0,0260	0,0804
Oribacterium.asaccharolyticum	0,0260	0,0804
Christensenella.sp.Marseille.P3954	0,0101	0,0497
Butyrivibrio.sp.AC2005	0,0260	0,0804
Anaerotruncus.sp.G3.2012.	0,0022	0,0384
Holdemania.filiformis	0,0048	0,0384
Catabacter.hongkongensis	0,0050	0,0384
Pseudomonas.putida	0,0442	0,1127
Firmicutes.bacterium.CAG.534	0,0087	0,0461
Lachnospiraceae.bacterium.AM48.27BH	0,0450	0,1127
Anaeromassilibacillus.senegalensis	0,0303	0,0883
Gordonibacter.urolithinfaciens	0,0123	0,0554
Dendrosporobacter.quercicolus	0,0200	0,0709
Blautia.schinkii	0,0022	0,0384

Clostridium.sp.AF27.2AA	0,0200	0,0709
Ruminiclostridium.sufflavum	0,0438	0,1127
Mordavella.sp.Marseille.P3756	0,0260	0,0804
Eggerthia.catenaformis	0,0159	0,0606
Propionispora.vibrioides	0,0442	0,1127
Lachnospiraceae.bacterium.V9D3004	0,0260	0,0804
Clostridium.sp.CAG.81	0,0200	0,0709
Clostridium.methylpentosum	0,0087	0,0461
Clostridiaceae.bacterium.JG1575	0,0496	0,1238
Parabacteroides.sp.AF14.59	0,0442	0,1127
Bacillus.subtilis	0,0124	0,0554
Ruminococcaceae.bacterium.CPB6	0,0152	0,0606
Ruminococcus.gnavus	0,0087	0,0461
Clostridiales.bacterium.TF09.2AC	0,0194	0,0709
Clostridiales.bacterium.41.12.two.minus	0,0087	0,0461
Clostridium.sp.Marseille.P3244	0,0411	0,1084
Eubacterium.callanderi	0,0100	0,0497
Blautia.sp.N6H1.15	0,0087	0,0461
Lachnospiraceae.bacterium.XBD2001	0,0081	0,0461
Coprococcus.comes	0,0043	0,0384
bacterium	0,0129	0,0554
Erysipelotrichaceae.bacterium.NK3D112	0,0159	0,0606
Clostridium.pasteurianum	0,0049	0,0384
Anaerocolumna.jejuensis	0,0087	0,0461
Clostridium.sp.AF15.17LB	0,0022	0,0384
Duncaniella.sp.C9	0,0260	0,0804
Clostridiales.bacterium.36.14	0,0411	0,1084
Roseburia.hominis	0,0043	0,0384
Firmicutes.bacterium.CAG.791	0,0081	0,0461
Clostridium.sp.OM02.18AC	0,0245	0,0804
Ruminiclostridium.cellulolyticum	0,0063	0,0433
uncultured.Lachnospiraceae.bacterium	0,0260	0,0804
Anaerocolumna.xylanovorans	0,0087	0,0461
Lachnospiraceae.bacterium.OF09.6	0,0161	0,0606
Clostridiales.bacterium.Firm.17	0,0260	0,0804
Anaerovibrio.lipolyticus	0,0200	0,0709
Oribacterium.parvum	0,0101	0,0497
Marvinbryantia.formatexigens	0,0022	0,0384
Firmicutes.bacterium.CAG.882	0,0152	0,0606
Lachnospiraceae.bacterium.Zagget13	0,0063	0,0433
uncultured.Firmicutes.bacterium	0,0158	0,0606
Lachnospiraceae.bacterium.KH1T2	0,0281	0,0867
Firmicutes.bacterium.CAG.65.45.313	0,0450	0,1127
Lachnospira.pectinoschiza	0,0260	0,0804
Clavaria.fumosa	0,0196	0,0709
Bariatricus.massiliensis	0,0152	0,0606
Butyrivibrio.hungatei	0,0260	0,0804
Alistipes.sp.CAG.831	0,0364	0,1028
Blautia.sp.CAG.52	0,0050	0,0384
Ruminococcus.sp.AF20.12LB	0,0367	0,1028
Ruminiclostridium.cellobioparum	0,0225	0,0788
Shuttleworthia.satelles	0,0247	0,0804
Ruminococcus.sp.Zagget7	0,0161	0,0606
Ruminococcus.sp.UNK.MGS.30	0,0043	0,0384
Ruminococcus.sp.CAG.379	0,0161	0,0606
Clostridium.sp.AF32.12BH	0,0049	0,0384
Eubacterium.sp.CAG.38	0,0022	0,0384
Ruminococcus.sp.CAG.403	0,0022	0,0384

Bacteroides.uniformis	0,0260	0,0804
Lachnoclostridium.sp.Marseille.P6806	0,0161	0,0606
Lachnospiraceae.bacterium.AB2028	0,0124	0,0554
Clostridium.scindens	0,0048	0,0384
Dorea.sp.5.2	0,0022	0,0384
Acetitomaculum.ruminis	0,0152	0,0606
Firmicutes.bacterium.OM08.11AC	0,0198	0,0709
Eubacterium.barkeri	0,0411	0,1084
Coriobacterium.glomerans	0,0161	0,0606
Bacteroides.sp.CAG.443	0,0247	0,0804
Herbinix.luporum	0,0411	0,1084
Enterorhabdus.mucosicola	0,0260	0,0804
Clostridium.sp.CAG.632	0,0152	0,0606
Clostridium.magnum	0,0245	0,0804
Paraeggerthella.hongkongensis	0,0292	0,0883
Butyrivibrio.sp.VCB2001	0,0081	0,0461
Lachnospiraceae.bacterium.XBB2008	0,0087	0,0461
Phascolarctobacterium.succinatutens	0,0301	0,0883
Clostridium.sp.BNL1100	0,0087	0,0461
Ruminococcus.sp.OM05.10BH	0,0087	0,0461
Proteiniclasticum.ruminis	0,0334	0,0971
Hydrogenoanaerobacterium.saccharovorans	0,0087	0,0461
Pseudobutyrivibrio.sp.YE44	0,0127	0,0554
Clostridium.leptum.CAG.27	0,0062	0,0433
Lachnospiraceae.bacterium.AC2014	0,0022	0,0384
Ruminococcus.sp.CAG.330	0,0080	0,0461
Butyrivibrio.proteoclasticus	0,0022	0,0384
Ruminococcus.sp.AF18.22	0,0161	0,0606
Johnsonella.ignava	0,0411	0,1084
Lachnospiraceae.bacterium.XBB1006	0,0022	0,0384
Paenibacillus.mucilaginosus	0,0364	0,1028
Butyrivibrio.sp.NC2002	0,0022	0,0384
Hungateiclostridium.thermocellum	0,0049	0,0384
Roseburia.sp.CAG.471	0,0022	0,0384
Lachnospiraceae.bacterium.G41	0,0043	0,0384
Alistipes.sp.Marseille.P5061	0,0152	0,0606
Lachnospiraceae.bacterium.MA2020	0,0022	0,0384
Geosporobacter.ferrireducens	0,0260	0,0804
Acetobacterium.sp.KB.1	0,0200	0,0709
Blautia.sp.OM07.19	0,0050	0,0384
Clostridium.botulinum	0,0103	0,0500
Roseburia.intestinalis	0,0022	0,0384
Anaerocolumna.aminovalerica	0,0022	0,0384
Clostridium.polysaccharolyticum	0,0022	0,0384
Butyrivibrio.sp.INlla14	0,0050	0,0384
Clostridium.sp.CAG.411	0,0295	0,0883
Peptoniphilus.ivorii	0,0115	0,0552
Clostridiales.bacterium.44.9	0,0301	0,0883
Lachnospiraceae.bacterium.GAM79	0,0161	0,0606
Butyrivibrio.sp.XBB1001	0,0022	0,0384
Christensenella.sp.AF73.05CM02	0,0450	0,1127
Eubacterium.ruminantium	0,0126	0,0554
Roseburia.sp.AM16.25	0,0200	0,0709
Coprococcus.eutactus	0,0450	0,1127
Cronobacter.malonaticus	0,0129	0,0554
Bacteroides.helcogenes	0,0303	0,0883
Roseburia.sp.CAG.303	0,0152	0,0606
Clostridium.sp.SY8519	0,0043	0,0384

Anaerostipes.hadrus	0,0022	0,0384
Catonella.morbi	0,0022	0,0384
Paenibacillus.pasadenensis	0,0159	0,0606
Caldicoprobacter.faecalis	0,0411	0,1084
Alistipes.finegoldii	0,0303	0,0883
Sphingobacteriaceae.bacterium	0,0442	0,1127
Roseburia.sp.TF10.5	0,0050	0,0384
Butyrivibrio.sp.VCD2006	0,0129	0,0554
Eubacterium.limosum	0,0152	0,0606
Faecalicatena.orotica	0,0050	0,0384
uncultured.Faecalibacterium.sp.	0,0161	0,0606
Clostridium.sp.CAG.590	0,0022	0,0384
Firmicutes.bacterium.AM59.13	0,0050	0,0384
Bacteroides.sp.OM08.11	0,0081	0,0461
Bacilliculturomica.massiliensis	0,0247	0,0804
Blautia.producta	0,0022	0,0384
Alistipes.indistinctus	0,0087	0,0461
Pseudobutyrivibrio.sp.49	0,0245	0,0804
Lachnospiraceae.bacterium.KHCPX20	0,0237	0,0804
Butyrivibrio.sp.CAG.318	0,0043	0,0384
Clostridiales.bacterium.KLE1615	0,0129	0,0554
Eggerthella.sp.YY7918	0,0123	0,0554
Lachnotalea.glycerini	0,0050	0,0384
Lachnospiraceae.bacterium.G11	0,0247	0,0804
Clostridium.cellulovorans	0,0047	0,0384
Fusarium.oxysporum	0,0060	0,0433
Brevibacillus.brevis	0,0442	0,1127
Eubacterium.ventriosum	0,0260	0,0804
Acetobacterium.bakii	0,0049	0,0384
Roseburia.sp.AM59.24XD	0,0050	0,0384
Lachnospiraceae.bacterium.oral.taxon.082	0,0129	0,0554
Alistipes.sp.Zagget8	0,0260	0,0804
Butyrivibrio.sp.Su6	0,0081	0,0461
Bacteroides.sp.AF39.11AC	0,0245	0,0804
Oribacterium.sp.NK2B42	0,0081	0,0461
Clostridium.kluyveri	0,0077	0,0461
Eubacterium.rectale.CAG.36	0,0043	0,0384
Ruminococcaceae.bacterium.AB4001	0,0090	0,0476
Clostridium.sp.ASF502	0,0022	0,0384
Lachnospiraceae.bacterium.C10	0,0357	0,1025
Butyrivibrio.sp.NC3005	0,0081	0,0461
Butyrivibrio.sp.MC2013	0,0081	0,0461
Pseudobutyrivibrio.ruminis	0,0043	0,0384
Traorella.massiliensis	0,0050	0,0384
Lachnospiraceae.bacterium.AC3007	0,0022	0,0384
Paenibacillus.ginsengihumi	0,0127	0,0554
Clostridium.populeti	0,0050	0,0384
Blautia.sp.Marseille.P3201T	0,0022	0,0384
Clostridiales.bacterium.41.21.two.genomes	0,0080	0,0461
Dielma.fastidiosa	0,0050	0,0384
Anaerobium.acetethylicum	0,0050	0,0384
Lachnospiraceae.bacterium.AC2028	0,0049	0,0384
Bacteroidales.bacterium.55.9	0,0081	0,0461
Lachnoanaerobaculum.sp.OBRC5.5	0,0152	0,0606
Alistipes.timonensis	0,0087	0,0461
Lachnospiraceae.bacterium.NE2001	0,0159	0,0606
Roseburia.sp.CAG.50	0,0064	0,0433
Clostridiales.bacterium.Firm.06	0,0364	0,1028

Paenibacillus.oryzae	0,0301	0,0883
Clostridium.sp.AM27.31LB	0,0260	0,0804
Treponema.primitia	0,0124	0,0554
Treponema.brennaborense	0,0420	0,1104
Butyrivibrio.sp.WCD3002	0,0048	0,0384
Eubacterium.sp.CAG.252	0,0260	0,0804
Faecalimonas.umbilicata	0,0050	0,0384
Solobacterium.moorei	0,0152	0,0606
Holdemanella.biformis	0,0081	0,0461
Butyrivibrio.sp.INlla16	0,0292	0,0883
Alistipes.sp.CAG.268	0,0152	0,0606
Ruminococcus.sp.Zagget11	0,0152	0,0606
Lachnoclostridium.phytofermentans	0,0048	0,0384
Desulfotomaculum.putei	0,0354	0,1021
Lachnospiraceae.bacterium.NLAE.zl.G231	0,0064	0,0433
Peptoanaerobacter.stomatis	0,0129	0,0554
Roseburia.sp.CAG.100	0,0245	0,0804
Methylomusa.anaerophila	0,0292	0,0883
Butyrivibrio.sp.MC2021	0,0022	0,0384
Lachnospiraceae.bacterium.P6A3	0,0124	0,0554
Roseburia.sp.CAG.182	0,0022	0,0384
Firmicutes.bacterium.CAG.227	0,0161	0,0606
Blautia.sp.AF19.10LB	0,0295	0,0883
Roseburia.sp.40.7	0,0049	0,0384
Blautia.massiliensis	0,0049	0,0384
Butyricicoccus.sp.AM28.25	0,0087	0,0461
Streptococcus.oralis	0,0087	0,0461
Blautia.sp.AF19.13LB	0,0022	0,0384
Butyrivibrio.sp.FCS006	0,0124	0,0554
Clostridium.sp.CAG.678	0,0247	0,0804
Anaerolineaceae.bacterium	0,0043	0,0384
Paenibacillus.sp.HW567	0,0124	0,0554
Gardnerella.vaginalis	0,0235	0,0804
Peptostreptococcaceae.bacterium.pGA.8	0,0063	0,0433
Clostridium.sp.DL.VIII	0,0100	0,0497
Clostridium.thermopalmarium	0,0185	0,0693
Pseudobutyrivibrio.sp.ACV.2	0,0022	0,0384
Clostridium.argentinense	0,0245	0,0804
Butyrivibrio.sp.MB2005	0,0301	0,0883
Clostridium.uliginosum	0,0376	0,1039
Ruminococcus.sp.CAG.382	0,0446	0,1127
Lachnospira.multipara	0,0129	0,0554
Paenibacillus.sp.NFR01	0,0370	0,1028
Xylaria.hypoxylon	0,0492	0,1230
Blautia.hydrogenotrophica	0,0081	0,0461
Oribacterium.sp.FC2011	0,0247	0,0804
Paenibacillus.sp.32O.W	0,0127	0,0554
Hungateiclostridium.cellulolyticum	0,0050	0,0384
Lachnospiraceae.bacterium.CAG.364	0,0022	0,0384
Butyrivibrio.crossotus	0,0081	0,0461
Hungateiclostridium.clariflavum	0,0049	0,0384
Paenibacillaceae.bacterium	0,0145	0,0606
Odoribacter.sp.AF15.53	0,0101	0,0497
Alistipes.sp.Marseille.P2431	0,0152	0,0606
Lachnospiraceae.bacterium.C6A11	0,0049	0,0384
Lachnospiraceae.bacterium.M18.1	0,0022	0,0384
Clostridium.sp.TF08.15	0,0292	0,0883
Fontibacillus.phaseoli	0,0157	0,0606

Pseudobutyrivibrio.xylanivorans	0,0192	0,0709
Enterococcus.pallens	0,0123	0,0554
Erysipelotrichaceae.bacterium.21.3	0,0022	0,0384
Dyadobacter.fermentans	0,0108	0,0523
Bacteroides.nordii	0,0129	0,0554
Anaerobutyricum.hallii	0,0087	0,0461
Eubacterium.yurii	0,0196	0,0709
Alistipes.finegoldii.CAG.68	0,0080	0,0461
Bacteroides.fluxus	0,0022	0,0384
Eubacterium.sp.CAG.274	0,0080	0,0461
Clostridium.mediterraneense	0,0379	0,1046
Alistipes.sp.58.9.plus	0,0081	0,0461
Vallitalea.sp.S15	0,0063	0,0433
Eubacterium.sp.CAG.603	0,0298	0,0883
Alistipes.sp.CHKCI003	0,0050	0,0384
Vibrio.parahaemolyticus	0,0064	0,0433
Eubacterium.sp.3.1.31	0,0127	0,0554
Anaerospora.hongkongensis	0,0047	0,0384
Pseudobutyrivibrio.sp.MD2005	0,0049	0,0384
Lachnobacterium.bovis	0,0022	0,0384
Desulfallas.gibsoniae	0,0194	0,0709
Firmicutes.bacterium.AM41.5BH	0,0124	0,0554
Clostridium.amylolyticum	0,0222	0,0782
Clostridium.clostridioforme.CAG.132	0,0196	0,0709
uncultured.Desulfobacterium.sp.	0,0338	0,0978
Bacillus.sp.FJAT.29814	0,0357	0,1025
Clostridium.sp.AF02.29	0,0050	0,0384
Roseburia.sp.CAG.197	0,0161	0,0606
Eubacterium.sp.CAG.786	0,0295	0,0883
Anaerotruncus.sp.CAG.528	0,0064	0,0433
Subdoligranulum.sp.AM23.21AC	0,0043	0,0384
Lachnospiraceae.bacterium.C7	0,0043	0,0384
Eubacterium.sp.CAG.248	0,0063	0,0433
Butyrivibrio.sp.XPD2002	0,0063	0,0433
Erysipelotrichaceae.bacterium.6.1.45	0,0129	0,0554
Butyrivibrio.sp.AE3006	0,0245	0,0804
Pezicula.radicicola	0,0365	0,1028
Clostridiales.bacterium.GWC2.40.7	0,0066	0,0448
Bacillus.foraminis	0,0114	0,0546
Romboutsia.timonensis	0,0459	0,1149
Clostridium.estertheticum	0,0376	0,1039
Firmicutes.bacterium.CAG.341	0,0047	0,0384
Clostridium.nexile.CAG.348	0,0222	0,0782
Sporosarcina.ureae	0,0126	0,0554
Ruminococcus.sp.Phil15	0,0050	0,0384
Roseburia.faecis	0,0050	0,0384
Bacillus.pseudomycoides	0,0181	0,0679
Clostridium.sp.CAG.253	0,0411	0,1084
Butyricimonas.virosa	0,0063	0,0433
Paenibacillus.agaridevorans	0,0077	0,0461
Melampsora.abietis.canadensis	0,0112	0,0541
Clostridium.sp.CAG.1219	0,0199	0,0709
Alistipes.sp.56.sp.Nov.56.25	0,0123	0,0554
Butyrivibrio.sp.AE2005	0,0072	0,0461
Chryseobacterium.shandongense	0,0055	0,0414
Serpentinicella.alkaliphila	0,0112	0,0541
Bacteroidales.bacterium.M13	0,0022	0,0384
Khelaifiella.massiliensis	0,0055	0,0414

Caloramator.quimbayensis	0,0222	0,0782
Clostridium.collagenovorans	0,0250	0,0804
Clostridium.sp.AF50.3	0,0047	0,0384
Paraliobacillus.sp.X.1125	0,0054	0,0412
Paenibacillus.donghaensis	0,0049	0,0384
Candidatus.Saccharibacteria.bacterium	0,0047	0,0384
Hathewaya.proteolytica	0,0072	0,0461
Erysipelotrichaceae.bacterium.SG0102	0,0042	0,0384
Ruminococcus.sp.CAG.563	0,0054	0,0412
Veillonella.magna	0,0095	0,0494
Mycoplasma.sp.CAG.776	0,0078	0,0461
Clostridium.sp.CAG.921	0,0078	0,0461
Thermoanaerobacterium.thermosaccharolyticum	0,0078	0,0461
Lactobacillus.johnsonii	0,0022	0,0384
Dethiosulfatibacter.aminovorans	0,0046	0,0384
Clostridium.sp.CAG.343	0,0251	0,0804
Faecalitalea.cylindroides	0,0099	0,0497
Clostridium.sp.CAG.245	0,0057	0,0425
Paenibacillus.naphthalenovorans	0,0042	0,0384
Eubacterium.sp.TM05.53	0,0061	0,0433
Romboutsia.ilealis	0,0099	0,0497
Enterococcus.mundtii	0,0097	0,0497
Enterococcus.termitis	0,0099	0,0497
Clostridiales.bacterium.oral.taxon.876	0,0043	0,0384
Propionibacterium.sp.	0,0049	0,0384
Bacillus.simplex	0,0043	0,0384
Clostridiales.bacterium.Firm.18	0,0036	0,0384
Clostridium.dakarense	0,0062	0,0433
Mitsuokella.sp.oral.taxon.131	0,0037	0,0384
Firmicutes.bacterium.CAG.582	0,0036	0,0384
Clostridium.aceticum	0,0036	0,0384
Variovorax.paradoxus	0,0037	0,0384
Turicibacter.sanguinis	0,0049	0,0384
Firmicutes.bacterium.AF36.3BH	0,0037	0,0384
Mailhella.sp.	0,0050	0,0384
Azospirillum.sp.TSO35.2	0,0023	0,0384
Bacteroides.sp.AR29	0,0027	0,0384
Clostridiales.bacterium.Firm.05	0,0027	0,0384
Tepidibacter.formicigenes	0,0027	0,0384
Thermoanaerobacterium.sp.RBIITD	0,0027	0,0384
Anoxybacillus.tepidamans	0,0028	0,0384
Bacteroides.sp.A1C1	0,0028	0,0384
Bacteroides.sp.AF39.16AC	0,0028	0,0384
Burkholderiales.bacterium.YL45	0,0028	0,0384
Firmicutes.bacterium.CAG.41	0,0028	0,0384
Lysobacter.enzymogenes	0,0028	0,0384
Turicibacter.sp.H121	0,0028	0,0384
Geobacteraceae.bacterium.GWC2.58.44	0,0090	0,0476
Blautia.sp.OM06.15AC	0,0095	0,0494
Lactobacillus.hominis	0,0095	0,0494
Romboutsia.hominis	0,0095	0,0494
Bacteroides.sp.D20	0,0096	0,0494
Firmicutes.bacterium.AM55.24TS	0,0096	0,0494
Lactobacillus.camelliae	0,0096	0,0494
Bacillus.sp.FJAT.45385	0,0284	0,0870
Deinococcus.aquatilis	0,0284	0,0870
Fusobacterium.mortiferum	0,0284	0,0870
Granulicatella.elegans	0,0284	0,0870

Sulfurimonas.sp.	0,0284	0,0870

Suppl. Table 4: Differential species abundance in fecal samples of DY and DO mice.

Species	*p*_Unadjusted	FDR
Ruminococcaceae.bacterium.AB4001	0,0177	1
Firmicutes.bacterium.AM59.13	0,0200	1
Deinococcus.aquatilis	0,0204	1
Cytophaga.xylanolytica	0,0232	1
Listeria.monocytogenes	0,0249	1
Hypholoma.sublateritium	0,0259	1
Erysipelotrichaceae.bacterium.6.1.45	0,0352	1
Clostridium.sp.AM43.3BH	0,0356	1
Paenibacillus.sp.HW567	0,0375	1
Pelotomaculum.thermopropionicum	0,0406	1
Clostridium.sp.AM09.51	0,0413	1
Firmicutes.bacterium.AF36.19BH	0,0421	1
Lutispora.thermophila	0,0471	1

Suppl. Table 5: Differential microbial pathway abundance in fecal samples of Y and O C57BL/6J mice.

Pathway	Y	O	Difference	FC	logratio	*p*_Unadjusted	FDR
PWY0-1241: ADP-L-glycero-β-D-manno-heptose biosynthesis	87	1	86	87,00	6,44	0,000	0,03
PWY-6527: stachyose degradation	145	190	-45	-1,31	-0,39	0,000	0,06
PWY-241: C4 photosynthetic carbon assimilation cycle NADP-ME type	271	376	-105	-1,39	-0,47	0,002	0,16
PWY0-845: superpathway of pyridoxal 5'-phosphate biosynthesis and salvage	307	3	304	102,33	6,68	0,002	0,16
THRESYN-PWY: superpathway of L-threonine biosynthesis	343	314	29	1,09	0,13	0,002	0,16
PWY0-162: superpathway of pyrimidine ribonucleotides de novo biosynthesis	278	123	155	2,26	1,18	0,002	0,16
PWY-7117: C4 photosynthetic carbon assimilation cycle PEPCK type	277	369	-92	-1,33	-0,41	0,003	0,21
PYRIDOXSYN-PWY: pyridoxal 5'-phosphate biosynthesis I	291	6	285	48,50	5,60	0,006	0,29
FUCCAT-PWY: fucose degradation	287	126	161	2,28	1,19	0,008	0,36
PWY-6606: guanosine nucleotides degradation II	61	110	-49	-1,80	-0,85	0,009	0,36
PWY-6545: pyrimidine deoxyribonucleotides de novo biosynthesis III	224	286	-62	-1,28	-0,35	0,009	0,36
PWY0-1298: superpathway of pyrimidine deoxyribonucleosides degradation	22	298	-276	-13,55	-3,76	0,010	0,36
COBALSYN-PWY: adenosylcobalamin salvage from cobinamide I	4	348	-344	-87,00	-6,44	0,015	0,47
PWY-6969: TCA cycle V (2-oxoglutarate:ferredoxin oxidoreductase)	7	362	-355	-51,71	-5,69	0,015	0,47
PWY0-1586: peptidoglycan maturation (meso-diaminopimelate containing)	241	319	-78	-1,32	-0,40	0,023	0,66
PWY-6608: guanosine nucleotides degradation III	97	138	-41	-1,42	-0,51	0,033	0,78
PWY0-862: (5Z)-dodecenoate biosynthesis I	48	359	-311	-7,48	-2,90	0,040	0,78
HEME-BIOSYNTHESIS-II: heme b biosynthesis I (aerobic)	312	4	308	78,00	6,29	0,040	0,78

PWY-7282: 4-amino-2-methyl-5-diphosphomethylpyrimidine biosynthesis II	323	4	319	80,75	6,34	0,040	0,78
P164-PWY: purine nucleobases degradation I (anaerobic)	62	102	-40	-1,65	-0,72	0,045	0,78
PWY-7383: anaerobic energy metabolism (invertebrates cytosol)	208	122	86	1,70	0,77	0,036	0,78

Suppl. Table 6: Differential microbial pathway abundance in fecal samples of DY and DO mice.

Pathway	DY	DO	Difference	FC	logratio	*p*_Unadjusted	FDR
PYRIDOXSYN-PWY: pyridoxal 5'-phosphate biosynthesis I	70	10	60	7,0	2,807	0,023	0,997
PWY-5723: Rubisco shunt	333	142	191	2,3	1,230	0,023	0,997
PWY-241: C4 photosynthetic carbon assimilation cycle NADP-ME type	77	15	62	5,1	2,360	0,026	0,997
PWY0-845: superpathway of pyridoxal 5'-phosphate biosynthesis and salvage	115	21	94	5,5	2,453	0,032	0,997
P162-PWY: L-glutamate degradation V (via hydroxyglutarate)	134	2	132	67,0	6,066	0,035	0,997
PWY-6470: peptidoglycan biosynthesis V	166	1	165	166,0	7,375	0,035	0,997

Suppl. Table 7: Targeted ALDEx2 analysis of microbial VB6 biosynthesis/salvage pathways in Y and O mice.

Pathway	Estimate (Log_2FC)	SE	*t*-value	*p*-value	*q*-value
PYRIDOXSYN-PWY: pyridoxal 5'-phosphate biosynthesis I	8,575	2,015	4,357	0,0020	0,322
PWY0-845: superpathway of pyridoxal 5'-phosphate biosynthesis and salvage	6,574	2,371	2,818	0,0214	0,971

Suppl. Table 8: Targeted ALDEx2 analysis of microbial VB6 biosynthesis/salvage pathways in DY and DO mice.

**Pathway**	**Estimate (Log_2FC)**	**SE**	***t*-value**	***p*-value**	***q*-value**
PYRIDOXSYN-PWY: pyridoxal 5'-phosphate biosynthesis I	6,878	1,921	3,600	0,0022	0,548
PWY0-845: superpathway of pyridoxal 5'-phosphate biosynthesis and salvage	6,789	1,887	3,634	0,0027	0,522
